# The nature and genomic landscape of repetitive DNA classes in *Chrysanthemum nankingense* shows recent genomic changes

**DOI:** 10.1093/aob/mcac066

**Published:** 2022-05-27

**Authors:** Fengjiao Zhang, Fadi Chen, Trude Schwarzacher, J S Heslop-Harrison, Nianjun Teng

**Affiliations:** Key Laboratory of Landscaping, Ministry of Agriculture and Rural Affairs, Key Laboratory of Biology of Ornamental Plants in East China, National Forestry and Grassland Administration, College of Horticulture, Nanjing Agricultural University, Nanjing 210095, China; Institute of Botany, Jiangsu Province and Chinese Academy of Sciences (Nanjing Botanical Garden Mem. Sun Yat-Sen), Nanjing, 210014, China; Department of Genetics and Genome Biology, University of Leicester, Leicester LE1 7RH, UK; Key Laboratory of Landscaping, Ministry of Agriculture and Rural Affairs, Key Laboratory of Biology of Ornamental Plants in East China, National Forestry and Grassland Administration, College of Horticulture, Nanjing Agricultural University, Nanjing 210095, China; Department of Genetics and Genome Biology, University of Leicester, Leicester LE1 7RH, UK; Key Laboratory of Plant Resources Conservation and Sustainable Utilization/Guangdong Provincial Key Laboratory of Applied Botany, South China Botanical Garden, Chinese Academy of Sciences, Guangzhou, 510650, China; Department of Genetics and Genome Biology, University of Leicester, Leicester LE1 7RH, UK; Key Laboratory of Plant Resources Conservation and Sustainable Utilization/Guangdong Provincial Key Laboratory of Applied Botany, South China Botanical Garden, Chinese Academy of Sciences, Guangzhou, 510650, China; Key Laboratory of Landscaping, Ministry of Agriculture and Rural Affairs, Key Laboratory of Biology of Ornamental Plants in East China, National Forestry and Grassland Administration, College of Horticulture, Nanjing Agricultural University, Nanjing 210095, China

**Keywords:** *Chrysanthemum*, genome organization, repetitive DNA, LTR retroelements, *k*-mer analysis, RepeatExplorer, fluorescent *in situ* hybridization

## Abstract

**Background and Aims:**

Tandemly repeated DNA and transposable elements represent most of the DNA in higher plant genomes. High-throughput sequencing allows a survey of the DNA in a genome, but whole-genome assembly can miss a substantial fraction of highly repeated sequence motifs. *Chrysanthemum nankingense* (2*n* = 2*x* = 18; genome size = 3.07 Gb; Asteraceae), a diploid reference for the many auto- and allopolyploids in the genus, was considered as an ancestral species and serves as an ornamental plant and high-value food. We aimed to characterize the major repetitive DNA motifs, understand their structure and identify key features that are shaped by genome and sequence evolution.

**Methods:**

Graph-based clustering with RepeatExplorer was used to identify and classify repetitive motifs in 2.14 millions of 250-bp paired-end Illumina reads from total genomic DNA of *C. nankingense*. Independently, the frequency of all canonical motifs *k*-bases long was counted in the raw read data and abundant *k*-mers (16, 21, 32, 64 and 128) were extracted and assembled to generate longer contigs for repetitive motif identification. For comparison, long terminal repeat retrotransposons were checked in the published *C. nankingense* reference genome. Fluorescent *in situ* hybridization was performed to show the chromosomal distribution of the main types of repetitive motifs.

**Key Results:**

Apart from rDNA (0.86 % of the total genome), a few microsatellites (0.16 %), and telomeric sequences, no highly abundant tandem repeats were identified. There were many transposable elements: 40 % of the genome had sequences with recognizable domains related to transposable elements. Long terminal repeat retrotransposons showed widespread distribution over chromosomes, although different sequence families had characteristic features such as abundance at or exclusion from centromeric or subtelomeric regions. Another group of very abundant repetitive motifs, including those most identified as low-complexity sequences (9.07 %) in the genome, showed no similarity to known sequence motifs or tandemly repeated elements.

**Conclusions:**

The *Chrysanthemum* genome has an unusual structure with a very low proportion of tandemly repeated sequences (~1.02 %) in the genome, and a high proportion of low-complexity sequences, most likely degenerated remains of transposable elements. Identifying the presence, nature and genomic organization of major genome fractions enables inference of the evolutionary history of sequences, including degeneration and loss, critical to understanding biodiversity and diversification processes in the genomes of diploid and polyploid *Chrysanthemum*, Asteraceae and plants more widely.

## Introduction

The genomes of plant and animal species include abundant repetitive DNA, sequence motifs of 2–10 000 or more bases that are repeated hundreds or even millions of times in the genome. It has been proved to play multiple roles in the genome, including genome size and stability, architecture, and modification of gene expression (Heslop-Harrison and Schwarzacher, 2011; Mehrotra and Goyal, 2014, Biscotti *et al.*, 2015; Wendel *et al.*, 2016). The amplification and contraction of repetitive DNA does have consequences for genome evolution, defining differences between genomes or species and often being the most rapidly evolving component of the genomes in both copy number and sequences (Biscotti *et al.*, 2015). Because of the much more rapid evolution of repetitive regions compared with low-copy or unique DNA sequences, the repetitive sequences can reveal the evolutionary history in short time scales (Negm *et al.*, 2021). Many studies of model organisms also proved that repetitive elements play important roles in many biological processes, including gene regulation of adaptive phenotype and epigenetic variation, mechanisms underlying reproductive isolation and speciation (Stuart *et al.*, 2016; Niu *et al.*, 2019; Schrader and Schmitz, 2019).

Repetitive sequences vary extensively in sequence and dispersion patterns, which are often categorized into dispersed transposable elements (TEs) and tandemly repeated (or satellite) sequences (Biscotti *et al.*, 2015). Typically, retrotransposons and their derivatives are the largest fraction of the genome and may be located over all or most of the chromosomes, but many of these divergent retrotransposons have yet to be categorized in Asteraceae (Xiong *et al.*, 2014). Tandemly repeated elements have also been identified in many species (representing several percent of the DNA), e.g. maize (Sharma *et al.*, 2013), wheat (Cheng and Murata, 2003), potato (Gong *et al.*, 2012), and oat (Liu *et al.*, 2019), with motif lengths representing DNA folding around one or two nucleosomes (140–360 bp, ~150 bp DNA for a single nucleosome spaced by a variable unwrapping linker region of ~30–60 bp) often occurring in blocks around centromeres or subtelomeric locations on chromosomes (Vershinin and Heslop-Harrison, 1998; Rao *et al.*, 2010; Heslop-Harrison and Schwarzacher, 2011, 2013).

In the past, repetitive sequences have been identified by screening genomic DNA clones, as restriction satellites, exploiting conserved motifs, or in sequence assemblies, by their structure and similarities to known repetitive DNA motifs. High-throughput sequencing surveys all the DNA in a genome, but whole-genome sequence assemblies tend to under-represent repeats as they are masked and often collapsed, so one repeat in the assembly represents hundreds of genomic copies, either in tandem or dispersed throughout the genome. Assemblies frequently end contigs with partial repeats as assembly cannot continue beyond this point (Lin *et al.*, 2016), or include repeats in a category of unassembled reads; thus repeats often remain unanalysed in reference genomes with the focus on genetic components of the genome. DNA sequence assembly algorithms have been optimized for generating long scaffolds of low-copy DNA, with *k*-mer and graph-based (Lin *et al.*, 2016) approaches that can also be applied to identify repeated motifs. Thus, novel algorithms for the identification of repetitive DNA in raw sequence reads have been developed recently, in particular analysing the abundance of all DNA short motifs of 16–150 bp (*k*-mer analysis), or using graph-based clustering (Novák *et al*., 2013, 2020). Novák *et al.* (2013) presented RepeatExplorer, which allows *de novo* repeat identification, and the cluster sizes provide a direct measure of the repeat proportion in the genome because the numbers of randomly generated reads are proportional to the genomic abundance of their corresponding sequences. RepeatExplorer has been used in many studies of diverse species for repeat identification (e.g. Macas *et al.*, 2015; Liu *et al.*, 2019; Vitales *et al.*, 2020; Jesionek *et al.*, 2021).

However, because of the challenges of multiple genomic locations and analysis of sequence data, chromosomal studies are necessary to examine the distribution and evolution of sequences. Fluorescent *in situ* hybridization (FISH) allows direct localization of DNA sequences on chromosomes, and repetitive DNA sequences usually generate characteristic FISH signals on individual chromosomes, which have proved informative to define genome structure, to trace species relationships, and in karyotyping (Schwarzacher and Heslop-Harrison, 2000; Jiang and Gill, 2006; Hemleben *et al.*, 2007). But no universal model of repeat structure across taxonomic groups has emerged, and analysis has tended to be selective for characterizable sequence motifs or those with variation amenable to assembly with algorithms optimized for low-copy sequences. So, the approach of combining bioinformatic analysis with cytology of *in situ* hybridization to chromosomes has been successfully used to quantify the genome repetitive landscape in many species, such as *Solanum* (He *et al.*, 2013), *Raphanus sativus* (He *et al.*, 2015) and *Avena* (Liu *et al.*, 2019).

*Chrysanthemum nankingense* (2*n* = 2*x* = 18; Asteraceae), considered to be an ancestral species in its genus (Neil, 2006; Ma *et al*., 2016, 2020), is grown as a high-value niche crop, an ornamental plant (ground cover and indoor flowering bushes) and a food (vegetable and flavouring, with traditional medicinal uses). In traditional Chinese medicine, chrysanthemum flowers were planted as herbal remedies as early as 1500 BC. It was considered to be a fragrant, cool and light herb, and benefits include improving the function of the cardiovascular system and lowering the levels of serum lipids (Wang and Xiao, 2013; Shahrajabian *et al.*, 2019). Whole-genome sequencing showed that the genome size is 3.07 Gb, including 69.58 % of repetitive elements, contributing to chrysanthemum’s genome size. The long terminal repeat (LTR) retroelements occupied the most abundant genome proportion (47.10 %), and there was also 17.62 % of unknown sequences. The content of DNA transposon and tandem repeats (satellites) was 3.18 and 0.32 %, respectively (Song *et al.*, 2018).

Considering that a large number of repetitive sequences may be masked during whole-genome assembly, here we used short reads of low-coverage genome sequences and aimed for comprehensive *de novo* identification of repeats in *C. nankingense* using RepeatExplorer and *k*-mer assemblies with no prior assumption about their nature, measuring the abundance and organization in the genome. Classification of identified repeats was then achieved by homology to known domains and sequences. The genomic distribution of repeats using some fragments of the cluster assemblies was verified and determined by FISH. Identifying the presence, nature and genomic locations of major genome fractions and insertion time estimation of LTR retroelements enables inference of genome and sequence evolutionary mechanisms and history, critical to understanding biodiversity and diversification processes in Asteraceae and plants more widely.

## MATERIALS AND METHODS

### Plant material and Illumina sequencing

The type specimen (no. NEAU0006698) is stored at the herbarium of Northeast Agricultural University (NEAU). *Chrysanthemum nankingense* seeds were planted in the chrysanthemum germplasm bank in China (Nanjing Agricultural University). DNA was extracted from young leaves of seedlings using the cetyltrimethylammonium bromide (CTAB) standard method (Porebski *et al.*, 1997). Total DNA was sequenced using the Illumina Hiseq 2500 platform with the PE250 strategy by the Beijing Genomics Institute (BGI) (http://www.genomics.org.cn/) (Shenzhen, Guangdong Province, China). The raw Illumina data (12.3 Gb) were submitted to GenBank with the BioProject ID PRJNA787776 and BioSample accession number SAMN23845234.

### Identification of repeat classes

The Galaxy platform running the program RepeatExplorer (Novák *et al*., 2013, 2020) was used to *de novo* identify highly repeated sequences in the genome from the raw reads. A total of 1.5 Gb (maximum limit 2 Gb) raw reads were uploaded to the website of RepeatExplorer. The reads were clustered into groups using a De Bruijn graph approach (Novák *et al*., 2013, 2020) under default parameters. Initial clusters were then connected through mates where there were extensive overlapping sequences. Clusters were classified using the automated Repeat Masker and Domain hits provided by RepeatExplorer (Viridiplantae) and used for percentage calculations for repeat classes and retroelement lineages (Supplementary Data Table S1). More often, some highly abundant sequences and also other clusters with high genome coverage were labelled as ‘low complexity’ or ‘simple repeat’; ‘low complexity’ is a term derived from re-association kinetic analysis of single-stranded DNA to identify the repetitive fraction (Flavell, 1982) and such sequences are often high copy motifs, while clusters labelled ‘simple repeat’ include sequences with extreme base-pair ratios on each strand rather than true simple sequence repeats or microsatellite motifs. The LTR_retriever v. 2.9.0 (Ou and Jiang, 2018) was performed to identify the LTR retroelements and their insertion times in the *C. nankingense* reference genome (Song *et al.*, 2018). The whole-genome data were downloaded from the *Chrysanthemum* Genome Database (Chrysanthemum_genome_scaffolds_v2.0.fasta, http://www.amwayabrc.com/download.htm; April 2020). The insertion time of LTR retroelements was calculated based on the nucleotide difference of the end of each intact LTR. Due to the special transposition and insertion mechanisms of LTR retroelements, the LTRs are initially highly homologous but their identity degenerates during time. First, all LTR retroelements were extracted using the biopython package, and TEsorter (Zhang *et al.*, 2019) was used to identify intact elements. Then the sequence identity difference between the LTRs at both ends was calculated (*d* = 100 % − identity %). According to the neutral selection theory, the time of transposon insertion into the genome can be calculated according to the formula *T* = *K*/2*μ* (Bowen and McDonald, 2001), where *K* is the genetic distance, which can be calculated according to the formula *K* = −3/4 × ln (1 − d × 4/3) in the Jukes–Cantor method (Kimura and Ohta, 1972). The number of base substitutions (*μ*) was taken as the average base substitution rate of rice, which was 1.3 × 10^−8^ in this study (Ma and Jackson, 2006). Finally, RepeatMasker was used to perform classification statistics on the whole-genome LTR elements of *C. nankingense*.

### k*-Mer analysis and assembly*

Many algorithms assemble the short sequences from *k*-mers for making contigs, and allow any size of *k*-mer for analysis. The complex repetitive DNA in several genomes has been quantized by the distribution of frequencies of long *k*-mers (20 ≤ *k *≤ 100) (Sindi *et al.*, 2008), so we used a wide range of *k* values from 16 to 128 for analysis in this study. The frequency of all canonical motifs *k* bases long was counted in the raw read data with *k*-mer sizes of 16, 21, 32, 64 and 128 using the program Jellyfish (Marcais and Kingsford, 2011). The most abundant fraction of *k*-mers was extracted from the data: 16-mer sequences repeated ≥10 000 and ≥50 000 times, 64-mer ≥10 000 and 1000 times, and 128-mer ≥1000 times. The 64-mers and 128-mers ≥1000 times were *de novo* assembled (Supplementary Data Table S2) using Geneious software (R10) (https://www.geneious.com/), with the medium sensitivity of Geneious assembler. Then, the assembled sequences were aligned with repetitive sequences identified by the program RepeatExplorer.

### Metaphase chromosome, probe preparation and FISH

Fresh root tips were fixed with 2 mm 8-hydroxyquinoline and ethanol/acetic acid fixation (3:1), then digested with proteolytic enzymes as described by Schwarzacher and Heslop-Harrison (2000). Individual root tips were transferred to a drop of 60 % acetic acid for metaphase chromosome preparation. Probes were generated by PCR from genomic DNA of *C. nankingense* template with primers (Supplementary Data Table S3) designed from contigs generated from RepeatExplorer clusters (abbreviated CL when describing the sequence in the cluster), *k*-mer motifs, or retroelements (*Copia* and *Gypsy*) (Flavell *et al.*, 1992*a*; Vershinin *et al.*, 2002). PCR was performed using a standard protocol (95 °C for 3 min, followed by 35 cycles of 95 °C for 30 s, primer-specific annealing temperature for 30 s, 72 °C for 45 s, and a final incubation at 72 °C for 1 min) and products were analysed by gel electrophoresis using 1 % agarose gels. To verify the sequence reliability, several PCR products of CL113Contig27, CL110Contig5 and CL122Contig35 were selected, cut from the gel and sequenced (SourceBioScience, Nottingham).

PCR fragments were labelled for FISH probes by biotin-16-dUTP or digoxigenin-11-dUTP (Roche Diagnostics, Basel, Switzerland) using the Bioprime Array CGH Genomic Labeling System (Invitrogen, Thermo Fisher Scientific, Waltham, MA, USA) according to the manufacturer’s instructions. Then, the processes of probe mixture preparation, probe and chromosomal DNA denaturation, hybridization and hybridization site detection were followed using the method described by Schwarzacher and Heslop-Harrison (2000) and Schwarzacher (2016) with small modifications. Probe mixtures were prepared with 50 % (v/v) formamide, 20 % (w/v) dextran sulphate, 2 × SSC (saline sodium citrate: 0.3 m NaCl, 0.03 m sodium citrate), 50–60 ng probe, 0.025 μg salmon sperm DNA and 0.125 % (w/v) SDS (sodium dodecyl sulphate) and 0.125 mm EDTA (ethylenediamine tetraacetic acid). Probe and chromosomal DNA were denatured together at 72 °C for 7 min on a Hybaid Omniblock (Thermo Fisher Scientific) and slowly cooled to 37 °C (which corresponds to 80 % stringency) and allowed to hybridize overnight. After washing, hybridization sites were detected with 2 µg/mL streptavidin conjugated to AlexaFluor594 (Molecular Probes, Thermo Fisher Scientific) and 4 µg/mL anti-digoxigenin conjugated to FITC (fluorescein isothiocyanate) (Roche Diagnostics). Slides were mounted in DAPI (4′,6-diamidino-2-phenylindole) antifade mixture and examined on a Nikon Eclipse N80i fluorescent microscope (Nikon, Tokyo, Japan) equipped with a DS-QiMc monochromatic camera (Nikon, Tokyo, Japan). Each metaphase was captured with three different filter sets (for AlexaFluor495, FITC and DAPI) and then the channels were overlaid and analysed with Adobe Photoshop CS6 (Adobe Systems, San Jose, CA, USA) using only cropping, and functions affecting the whole image equally. In total 14 clusters were FISHed and a minimum of two complete metaphases were analysed in detail for each cluster.

## RESULTS

### Repeat characterization

**Fig. 1.**
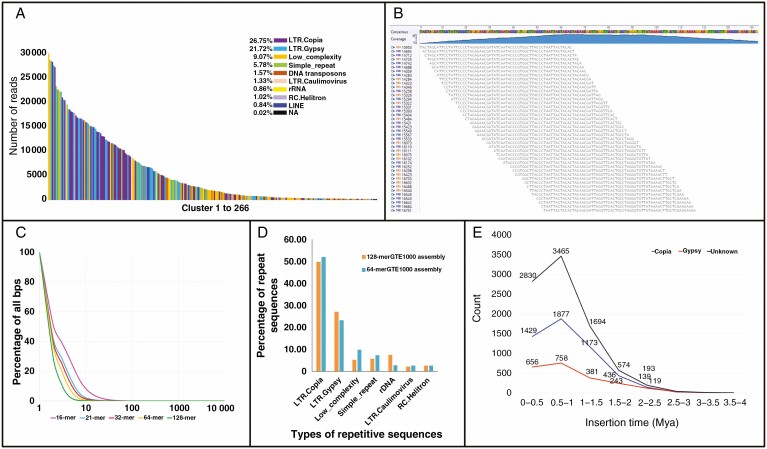
Repeat characterization of *C. nankingense* by graph-based clustering of similar reads in RepeatExplorer, analysis of the most frequent *k*-mer sequence motifs, and LTR_retriever analysis. (A) Identification and genome proportion of 266 clusters assembled with graph layout in RepeatExplorer output (for cluster details see Supplementary Data Table S1). (B) 64-mer GTE (greater than or equal to) 10 000 assembly using Geneious assembler. (C) Selected *k*-mer frequencies as a percentage of the whole *C. nankingense* genome. (D) Frequency of 64- and 128-mer assembled contigs as mapped to RepeatExplorer repetitive clusters. (E) Insertion time distribution of intact LTR retroelements in the whole genome of *C. nankingense*.

Both the analysis of graph-based clustering of similar sequences using the program RepeatExplorer and analysis of high-frequency *k*-mers enabled characterization *de novo* of the most abundant repetitive DNA sequence motifs and classes (Fig. 1) without bias from sequence assembly. A total of 2 136 022 paired-end 250-bp raw reads from genomic DNA of *C. nankingense* were clustered using RepeatExplorer. As a result, 68.96 % of the genome was identified as highly repetitive. After analysis of the graph layout in the RepeatExplorer output, a total of 266 clusters with each >0.01 % of the genome were found, and many were subsequently identified by comparison with known protein and other sequence motifs (Supplementary Data Table S1). LTR retroelements occupied the highest proportion of the genome and included 26.75 % *LTR.Copia*, 21.72 % *LTR.Gypsy* and 1.33 % as *LTR.Caulimovirus* superfamilies. Fewer than 16 % were classified as ‘low complexity’ and ‘simple repeat’, including some tandemly repeated motifs, and a further 1.6 % were DNA transposons (Fig. 1A, Supplementary Data Table S1).

For the *k*-mer analysis, selected values of *k* between 16 and 128 were used to search for their frequency within the genome (Fig. 1C; Supplementary Data Table S2). For each value, canonical *k*-mers that were present abundantly were extracted from the count data, and assembled to generate longer contigs and using a genome-walking strategy to identify the full-length repetitive motifs (Fig. 1B). To avoid analysis of any artefacts (such as sequencing primers or excessive hybrid molecules), we checked that the counts of each *k*-mer assembled across a contig were similar and represented both forward and reverse directions in the reads. Abundant motifs were classified using automated and manual comparisons with GenBank, retroelement domains and simple sequence repeats (SSRs). Further, sequences assembled by RepeatExplorer were aligned with contigs assembled by 64- and 128-mer showing that nearly half of the *k*-mer-assembled contigs mapped to the *LTR.Copia* superfamily and ~25 % were in the *LTR.Gypsy* superfamily (Fig. 1D). Other types of *k*-mer assembly included rDNA, *LTR.Caulimovirus* and *RC.Helitron*, each class <10 % consistent with frequencies found in RepeatExplorer (compare Fig. 1A and D). Overall, *k*-mer analysis found a higher proportion of LTR retroelements, but fewer other abundant repetitive types in the genome. Neither analysis was designed to identify short repeats such as the simple sequence repeats (SSRs or microsatellites) within low-copy regions used as molecular markers.

**Fig. 2.**
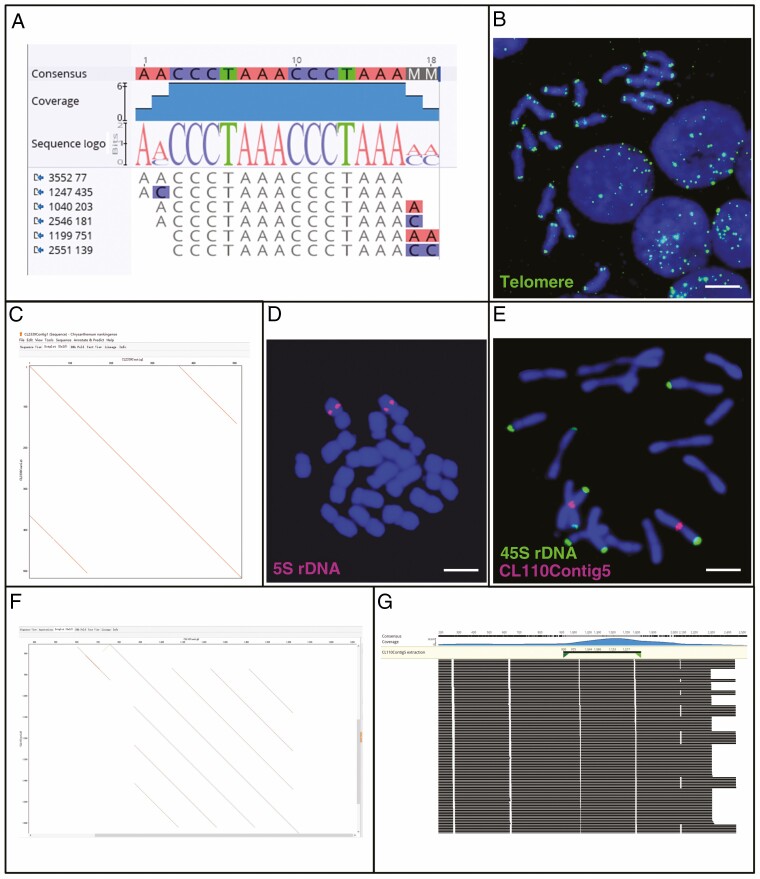
Characterization and genome location of tandemly repeated DNA sequences. (A) Telomere sequence assembly that was identified by 16-mer analysis. (B) FISH with the telomere sequence (green) to *C. nankingense* metaphase chromosomes and interphase nuclei (blue). Signal is visible as double dots at all chromosome ends and a few intercalary positions. At interphase, telomeres cluster at one side of the nucleus. Scale bar = 10 μm. (C) Self-dot-plot of 5S sequence (CL2339Contig1 in RepeatExplorer output). The repeat monomer of 363 bp is identified by the distance between the parallel lines. (D) Location of 5S rDNA (red) on the long arm of a pair of chromosomes (blue) of *C. nankingense* (2*n* = 18). Scale bar = 10 μm. (E) Chromosome location of 45S rDNA (eight green terminal signals) and the tandem repeat sequence RepeatExplorer CL110Contig5 (two red signals near the centromere). Scale bar = 10 μm. (F) Self-dot-plot of Cl110Contig5 to show the tandem repeat structure with a monomer of 150 bp. (G) Raw reads aligned to the consensus sequences of extracted CL110Contig5.

To verify and determine the chromosomal distribution of repeats, some fragments of the cluster assemblies were amplified by PCR from genomic DNA and verified by FISH (Figs 2 and 4–6 and Supplementary Data Fig. S1). The PCR confirmed that the repeats identified in the clustered short-read or *k*-mer assemblies were present in the genomic DNA and were not artefacts of the informatics approaches. The primers amplified regions of the expected length (Supplementary Data Table S3; for an example see Supplementary Data Fig. S1), and lack of smears demonstrated that there were specific structures in the genome associated with the primer pairs and we were not analysing a degenerate pool of sequences with some similarities.

**Fig. 3.**
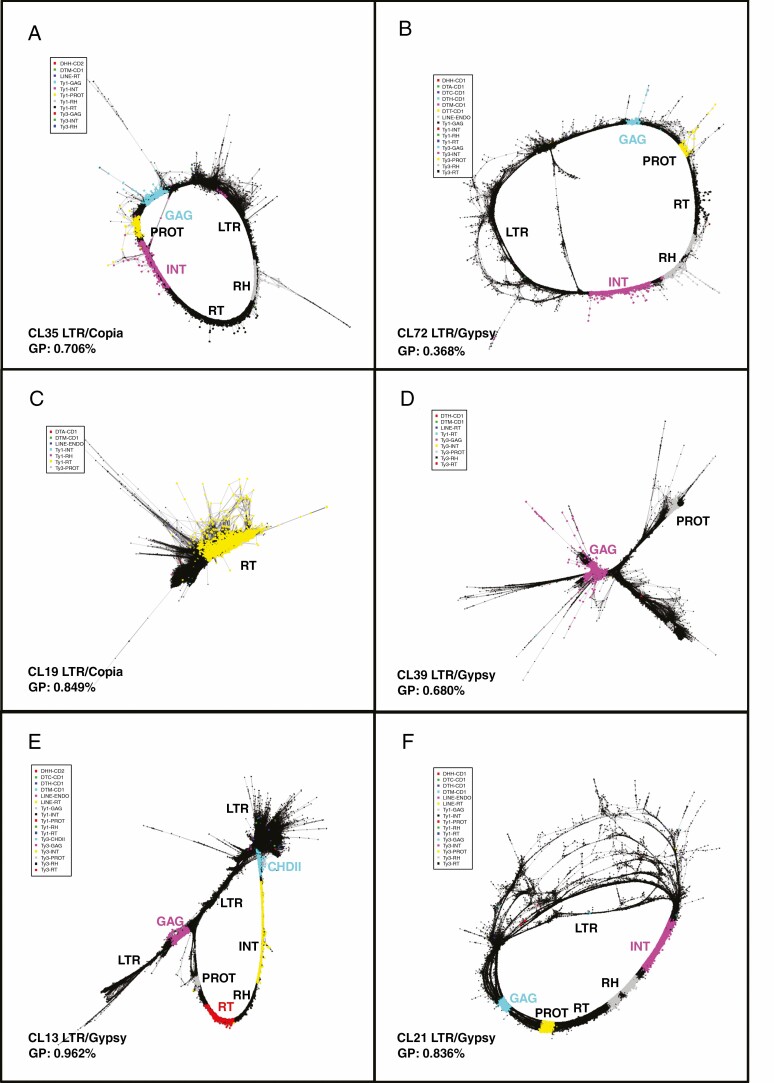
Typical graph shapes of LTR retroelements with most protein domains present. (A, B) Typical circular graphs with intact protein domains in the expected order of CL35 (*LTR.Copia*) and CL72 (*LTR.Gypsy*). The two LTR sequences fall together, indicating high homology and producing the circular shape. (C, D) Dense star shape with one or two domains and flanking sequences of CL19 (*LTR.Copia*) and CL39 (*LTR.Gypsy*). (E) The most abundant *LTR.Gypsy* superfamily with a circular shape and one more CHDII domain. The two LTRs are separated, indicating low or no homology. (F) Circular *LTR.Gypsy* superfamily with intact domains and divergent LTRs. INT, integrase; RH, ribonuclease H; RT, reverse transcriptase; PROT, protease; GAG, gag-polyprotein; CHDII, chromovirus chromodomain II; GP, genome proportion.

**Fig. 4.**
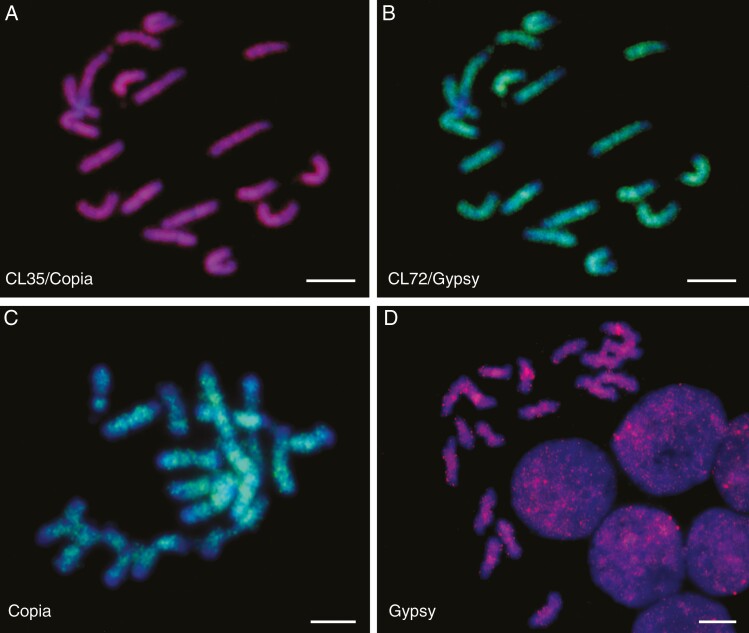
LTR retroelement distribution on chromosomes of *C. nankingense* (2*n* = 18) using FISH and DAPI staining (blue). (A) Chromosome-wide distribution of *Copia* CL35Contig138 (magenta). (B) The same metaphase as (B) with *Gypsy* CL72Contig70 (green), also showing distribution along the whole chromosomes but also some stronger signal at centromeres and some intercalary positions. (C, D) Distribution patterns of retroelements amplified from genomic DNA using universal primers for *Copia* (C, green) and *Gypsy* (D, magenta). Scale bar = 10 μm.

**Fig. 5.**
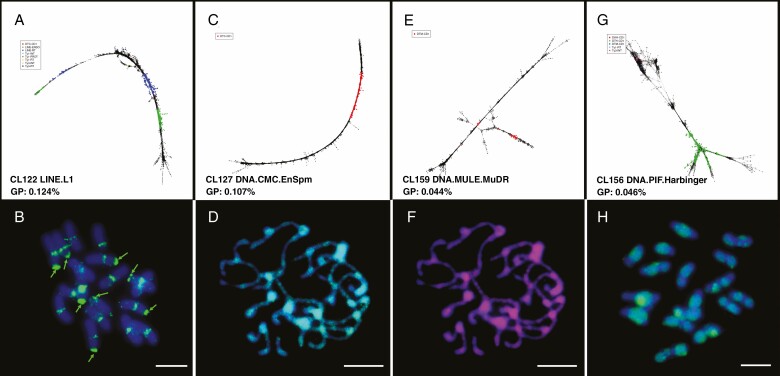
Graph layout and chromosome location of non-LTR retrotransposon and DNA transposon RepeatExplorer clusters in *C. nankingense*. (A, C, E, G) Linear graphs of CL122 (LINE), CL127 (DNA.CMC.EnSpm), CL159 (DNA.MULE.MuDR) and CL156 (DNA.PIF.Harbinger), respectively. GP, genome proportion. (B, D, F, H) FISH to chromosomes of the above four clusters. The non-LTR retrotransposons (LINEs) show centromeric sites and locate at the four pairs of rDNA sites (B, green arrows), while the three DNA transposon clusters (D, green signal; F, magenta signal; H, green signal) show signal dispersed over all chromosomes but missing some terminal regions. Scale bar = 10 μm.

**Fig. 6.**
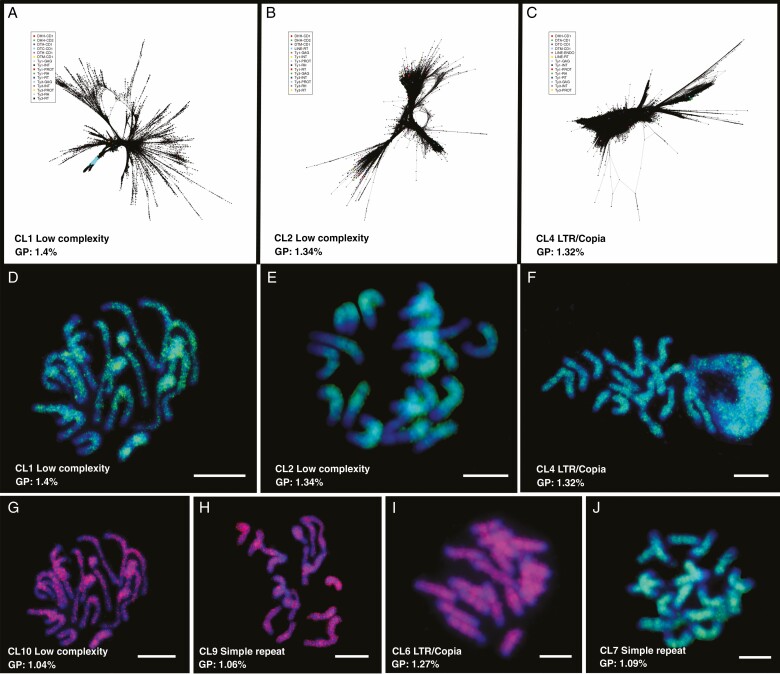
Graphs and chromosomal location of abundant low-complexity or unknown clusters in *C. nankingense*. (A–C) Graphs of low-complexity (CL1 and CL2) clusters and *LTR-Copia* cluster with very low domain hits (CL4). (D–F) Chromosome location of CL1Contig456, CL2Contig586 and CL4Contig81 showing dispersed distribution and higher concentration of proximal signal and less strong signal near the telomeres (E). (G–J) Chromosome location of four further abundant clusters in the top ten clusters; they are CL10Contig254, CL9Contig290, CL6Contig83 and CL7Contig38, respectively. Dispersed signal along chromosome arms with lack of signal in distal regions is visible (I). Scale bar = 10 μm. GP, genome proportion.

### Tandemly repeated DNA sequences

Both *k*-mer and RepeatExplorer identified three universal tandemly repeated sequences: the telomere, 45S and 5S rDNA (Fig. 2). In 16-mer analysis, there were millions of counts of ‘CCCTAAA’ (Fig. 2A), the telomere repeat sequence, located at chromosome ends by FISH (Fig. 2B). In RepeatExplorer output, we found the 363-bp long 5S rDNA monomer (CL2339Contig1, with <0.01 % of the genome; Fig. 2C). FISH with the PCR-amplified 5S sequence showed sites as double dots in the middle of the long arm of one chromosome pair (Fig. 2D). Three clusters (CL51, CL99, CL126) contained 45S rDNA, as a tandemly repeated 5842-bp long sequence including 18S, 5.8S and 26S rRNA genes and intergenic spacers, and localized in the subtelomeric region on four chromosome pairs (Fig. 2E); 45S rDNA represented 0.86 % of the reads. DNA sequences of 5S and 45S rDNA monomer were submitted to NCBI with the GenBank accession numbers MZ976787 and OK181863. CL110Contig5 is a tandem repeat with FISH signal close to the centromere on one pair of chromosomes that also had 45S rDNA signals (Fig. 2E). The sequence dot-plot of CL110Contig5 showed a structure with a 150-bp tandem repeat unit (Fig. 2F), supported by the raw reads of ~1.6 monomer copies, 0.16 % of the genome (Fig. 2G). In comparison, the whole-genome reference assembly (Song *et al.*, 2018) identified 0.32 % satellite content in the *C. nankingense* genome (Supplementary Data Table S4), less than the total of 1.02 % we have found as expected from analysis of raw reads rather than an assembly.

### LTR retroelements

Half of the RepeatExplorer clusters and *k*-mer assemblies included abundant sequences with homology to domains of LTR retroelements (Fig. 1A, D). These LTR retroelement sequences were classified into distinct lineages based on differences in structural and sequence features of the elements (Neumann *et al.*, 2019). For *LTR.Copia* elements, eight lineages and one unclassified group (3.79 % of the genome) were found (Supplementary Data Fig. S2A), with the Maximus/SIRE sequences most abundant (12.22 %). The *LTR*.*Gypsy* group included four lineages (*Athila*, 7.76 % of the genome; *Chromovirus*, 6.12 %; *Ogre/Tat*, 1.70 %; *Ivana/Oryco*, 0.60 %) and an unclassified group (5.54 %) (Supplementary Data Fig. S2B).

The principle of graph-based clustering organizes the sequence reads in a graph structure, where single reads are represented by vertices (nodes) and the edges are their sequence overlaps. Graph layouts in structure were formed by corresponding algorithms and labelled to distinguish different classes of repeats (Novák *et al.*, 2010). For example, the cluster-assembly graphs (Fig. 3) illustrate the greater conservation (superimposed nodes) of transcribed retrotransposon domains gag and pol and the variable divergence of the LTRs (spreading nodes and edges or even separated). There are more copies of the LTRs than the retrotransposon genes, representing solo LTRs left behind after transposon excision events, but some, due to their sequence homology, are still found associated within the full-length retroelement clusters. Further, the graphs reveal the relation of the left and right LTRs in each cluster; e.g. circular, lines and stars (Fig. 3). CL35 and CL72 were typical circular graphs, indicating the close homologies of the two LTRs, and also showed intact protein domains indicative of relatively recent integrations. Whereas CL35 consisted of the gene order GAG, PROT, INT, RT and RH, typical of *LTR.Copia* elements (Fig. 3A), CL72 had the gene order GAG, PROT, RT, RH and INT, typical of *Gypsy* elements (Fig. 3B). Other examples of very abundant retroelement clusters (under the clustering parameters chosen) show high rates of degeneracy and include only one or two protein domains and flanking sequences, such as CL19 (*LTR*.*Copia*) with abundant RT domains, and CL39 (*LTR.Gypsy*) with GAG and PROT domains (Fig. 3C, D).

The most abundant non-compound retroelement cluster was a Chromovirus (*LTR.Gypsy* superfamily; CL13, 0.962 %; Fig. 3E). Unlike other *Gypsy* families, it contained a well-defined CHDII domain that was next to INT (Hansen and Heslop-Harrison, 2004) and linked to the LTR region; the separate star-like shaped LTRs indicate that this cluster is an old element. CL21, an Ogre/Tat *Gypsy* element (Fig. 3F), is another specific circular LTR cluster with divergence. With the structural features, functional domains and sequence variability, but also high abundance of retroelements, the linear graphs indicate multiple sequence relationships between clusters; this was particularly apparent when our analysis also included the links between clusters provided by RepeatExplorer.

The first-pass graph-based clusters (Supplementary Data Table S1 and Supplementary Data Fig. S3) can sometimes be linked to other clusters by their structural features and sequences. In the case of LTR retroelements, this generates robust circular graphs with the gene domains and larger, more variable, domains with additional coverage and variability shown in the LTR region from intact and deleted (solo LTR) elements. The links are further confirmed by inclusion of paired-end reads between two clusters. The approach to resolving connections generated robust LTR retroelement clusters with all genes present. For example, one *LTR.Copia* element family member is connected by CL27 (with domain order GAG, PROT and INT), CL59 (RT and RH domains) and CL68 (GAG domain and several LTR-related sequences), suggesting an abundance of solo LTRs for this retroelement (Supplementary Data Figs S3 panel 8 and S4A). Additional clusters could also be fused to define different members of the *LTR.Copia* family by circular graphs [CL27-GAG linked to CL59-RH, and CL68 contigs were considered as LTR sequences in an *LTR.Copia* retrotransposon; and CL44 (POL) links to CL71 (GAG) and CL102 (LTR)] (Supplementary Data Figs S3 panel 10 and S4B).

According to the graph layout obtained by RepeatExplorer, the clusters with typical and complete characteristics were selected as candidate clusters for FISH to chromosomes, and the sequence of the contig with highest coverage in each cluster was extracted, used for primer design and amplified by PCR from genomic DNA to be used as probe for FISH. The results showed a generally dispersed distribution on chromosomes, with often less signal towards the end of the chromosomes (Fig. 4). Some unique characteristics are present and signal strength is roughly related to abundance as estimated by RepeatExplorer. Of the major LTR retroelement clusters, CL35 (*LTR.Copia*) was uniformly distributed along the chromosome arms with gaps at the centromeres (Fig. 4A); CL72 (*LTR.Gypsy*) was also dispersed, but more concentrated on the pericentromeres and some intercalary positions (Fig. 4B). These two contrasting distribution patterns are similar to those with *Copia* or *Gypsy* fragment pools amplified from genomic DNA using universal primers (Flavell *et al.*, 1992*b*; Vershinin *et al.*, 2002) and are shown in Fig. 4C, D.

In the reference genome of *C. nankingense* (Song *et al.*, 2018), 1 408 164 566-bp LTR retroelements were identified by LTR_retriever; they included 477 155 088 bp (18.88 % of the genome) *LTR*.*Copia*, 302 882 512 bp (11.98 %) *LTR.Gypsy* and a large number (628 126 966 bp; 24.85 %) of unknown elements, in total accounting for 55.72 % of the genome (Supplementary Data Table S4). We identified 107 429 843 bp of intact LTR retroelements, 4.25 % of the genome and <10 % of the total retroelements. The insertion times of the intact LTR retroelements were calculated (Fig. 1E), and showed that most concentrated around 0.5–1 million years ago (Mya), suggesting specific activities of transposition at that time.

### Non-LTR retrotransposons and DNA transposons

LINEs (non-LTR retrotransposons) (Schmidt, 1999) contributed 0.84 % of the *C. nankingense* genome (Fig. 1A). CL122 was the typical linear graph repeat, with a LINE-RT and LINE-ENDO domain (Fig. 5A), and showed centromeric sites as well as four pairs of rDNA sites after FISH (Fig. 5B, green arrows). DNA transposons (class I TEs; Biscotti *et al.*, 2015) accounted for 1.57 % (Fig. 1A): CL127, CL159 and CL156 included EnSpm, MULE and Harbinger families (Fig. 5C, E, G). Further, transposon Helitron-like domains (Xiong *et al*., 2014, 2016), not found before in *C. nankingense*, were 1.02 % of the genome. Together, these accounted for 2.59 % transposons in the genome, which is slightly less than the reported genome proportion of 3.2 % in the whole-genome sequence of *C. nankingense* (Song *et al.*, 2018; Supplementary Data Table S4)*. In situ* hybridization showed that DNA transposons such as CL127, CL159 and CL156 were distributed over all chromosomes, but less in some terminal regions (Fig. 5D, F) or the centromere (Fig. 5H).

### Additional sequence motifs: ‘repeats of unknown origin’

The 11 most abundant RepeatExplorer clusters (Supplementary Data Table S1), each represented >1 % (1.04–1.4 %) of the *C. nankingense* genome and together made up 13.2 % of the genome. The reads were assembled to diverse graph shapes, including low and high density or elongated star shapes (Fig. 6A–C). Homology to known sequences was not clear, and very few hits to TE domains were found. Automated annotation in RepeatExplorer labelled some as belonging to *LTR*.*Copia* or *LTR*.*Gypsy*, but the hits were extremely low (Supplementary Data Table S1), such that it does not represent a convincing identification.

To understand the nature of these unidentified sequences, often labelled ‘low complexity’ sequences in the RepeatExplorer output (Supplementary Data Table S1) but with abundant sequence motifs, we carried out FISH and investigated possible links and motif similarities between the clusters. The FISH signal of selected sequences from contigs within CL1, CL2, CL4, CL6, CL7, CL9 and CL10 was dispersed along all chromosomes with some being less at centromeres or the end of the chromosomes (Fig. 6D–J), and confirmed that the sequence motifs are indeed highly abundant within the genome. Refinement of the homology search of the NCBI GenBank database and the Viridae Plantae TE library identified some low-identity homology between our major unknown repeats and a series of sequences identified as a microsatellite library from *Chrysanthemum *×* morifolium* (Fan *et al.*, 2019). While our clusters did not include microsatellite motifs, these search results indicate that this class of sequences may be associated with dispersed genetic markers in the chrysanthemum genome.

Investigating the graph-based clustering parameters further, we found some sequence relationships between these abundant clusters: for example, CL2 has reads linking to CL6 (1.27 % of the genome), with CL6 linking to CL18 (0.868 %). They do not show any recognizable retroelement or coding domains in fragments more than 6.3 % (CL2, CL3, CL4, CL6 and CL10) of the genome, and show 7.9 % of the clustered reads at the end of clusters repeat motif). Because of the paired-end reads, these linked clusters were restructured (cut-off = 0.1) in different groups. A total of 16 groups contained at least three clusters (Supplementary Data Fig. S3). In group 2, it was a graph circle composed of CL2, CL7, CL10, CL16, CL22, CL45, CL46, CL58, CL65 and CL67, which was inferred to be a connected series of many LTR fragments.

## Discussion

In eukaryotes, TEs are a major genome component and have been proven to be an important source of variation in natural selection for evolving species or agronomic selection for interesting varieties (Quesneville, 2020). Generally, TEs are thought to insert anywhere in the genome, but some families exhibit striking deviations from a random distribution and different accumulation among chromosomal regions (Wright *et al.*, 2003). In many species, LTR retrotransposons are dominantly abundant in pericentromeric regions (Heslop-Harrison and Schwarzacher, 2011); in particular the *Athila* elements are almost exclusively inserted in the pericentromeric regions of *Arabidopsis thaliana*, but considerably less in the chromosome arms (Pereira, 2004; Quesneville, 2020). In this work, we characterized all the major repetitive DNA motifs in the 3.07 Gb *C. nankingense* genome by analysis of 12.3 Gb of short-read sequences, extensive bioinformatic analysis including two complementary, independent *de novo* repeat finding tools, sequence comparisons (Figs 1 and 3), and FISH (Figs 2 and 4–6) to show their abundance and genomic organization. Most reports of the nature and organization of the major repetitive DNA sequences in plants and animals (Biscotti *et al.*, 2015) discuss the presence of tandemly repeated sequences at several well-defined loci, along with TE families that are more dispersed over the genome but may be more abundant or excluded from particular domains. Another group of repeats, microsatellites or SSRs are typically too short (motifs <10 bp and copy number <20) to be characterized with the *de novo* prediction informatics tools used here unless in very long arrays. For example, the telomeric sequence (TTTAGGG)_*n*_, typical of plant species, is abundant and present at the ends of all chromosomes (Fig. 2A, B).

In many species, repetitive DNA sequences have been identified from cloned fragments or in sequence assemblies with high abundance (Heslop-Harrison and Schwarzacher, 2011; Mehrotra and Goyal, 2014, Wendel *et al.*, 2016). However, these motifs are often ignored in the analysis of large-scale genome organization or assumed to be related to retroelements. As an example from previous work in *Crocus*, Frello and Heslop-Harrison (2000) described several abundant clones that are useful for studying the phylogeny of species because of their differential amplification between species, but they had no homology to known sequences. For many species, whole-genome sequence assemblies are now available, but repetitive DNA causes problems in assembly. This occurs particularly with highly diverse sequences, or with tandemly repeated sequences where the junction fragments are linked without defining the tandem array between junctions, unless arrays are bridged using long-range sequencing technologies or mate-pair libraries (where the ends’ sequences can span repeat motifs). More recently, longer read technologies such as PacBio and Oxford Nanopore have been used to span arrays, but the high error rates mean high-accuracy Illumina short reads need to be used to correct these errors (Belser *et al.*, 2018; Wang *et al.*, 2021). Error correction is straightforward for single-copy DNA, where multiple short reads can be mapped to correct errors, but this correction approach does not allow the study of sequence variation in repeat arrays.

In *C. nankingense*, the whole genome is 37.2 % GC, while repeat cluster CL1 is 31.6 % GC; the average GC content of all repeat clusters is 36.1 %, showing a small difference from the whole genome. In this study, CL1 (annotated as low complexity in RepeatMasker) was linked to two *Gypsy* clusters, CL26 and CL79 (Supplementary Data Fig. S3 panel 6), suggesting that they may be the flanking long terminal repeat sequences of the *Gypsy* family. These flanking sequences not only allow the identification of LTR retroelements in the genome, but also play an important role in LTR retroelement life history. Because they contain regulatory motifs and are prone to ectopic recombination, the two flanking LTRs are the most characteristic feature of LTR retroelement insertions (Schulman, 2013). GC content was considered a distinctive feature of TE lineages, which is associated with methylation levels as quantitative variation in GC and methylation affects TE survival and proliferation (Stritt *et al.*, 2020). Here, given the widespread use of sequence reads as obtained here for assembly of genome sequences, there is little evidence for differential sequence representation in the reads. So, because of the high genome proportion and high GC content of CL1, we infer that methylation events might have happened frequently in the past and might have favoured the evolution and amplification of *Gypsy* elements in *C. nankingense*.

The FISH results confirmed the abundant presence of repeat sequences in the *C. nankingense* genome. In this study, RepeatExplorer generated a total of 266 cluster graphs with genome proportion >0.1 %, and found all abundant repetitive DNA families, which represented 68.96 % of the genome, which corresponds to the proportion found in the *C. nankingense* assembly by Song *et al.* (2018). Based on the structural features of the cluster graphs and the arrangement and homology of protein domains, RepeatExplorer clusters repeats could be classified to subfamilies and lineages and sequence variability was evident. The rDNA, known as tandemly repeated motifs in all species (e.g. Heslop-Harrison and Schwarzacher, 2011; Goffová and Fajkus, 2021), were revealed in the analyses: the 5S rDNA monomer (363 bp long but <0.01 % of the genome) was present at two pairs of sites while the 45S rDNA was more abundant (0.86 % of the genome) and present at four pairs of sites (Fig. 2C–E). Compared with many other species (e.g. Heslop-Harrison and Schwarzacher, 2011; Liu *et al.*, 2019), *Chrysanthemum* had relatively few tandem repeats, with none found at centromeres or in subtelomeric regions.

Some studies have explored the evolutionary mechanisms of copy number and chromosomal distribution of retrotransposons, including suppression of transposition or elimination of insertions, and non-random distribution along the chromosomes. The large accumulation of TEs close to the centromere in *A. thaliana* has been explained by non-random genomic distribution due to both selection against insertions in euchromatin and preferential targeting of heterochromatin that also limited the contribution of retrotransposon DNA to genome size expansion (Wright, 2003; Pereira, 2004). LTR retroelements represented 49.53 % of the *C. nankingense* genome. Most showed widespread distribution over chromosomes (Fig. 4), indicative of their dispersed nature (Biscotti *et al.*, 2015); each family had characteristic features, such as abundance or exclusion from centromeric or subtelomeric regions despite their common mode of amplification and dispersion. Our analysis also found that non-LTR retroelements and a LINE.L1 element showed discrete loci on chromosomes (Fig. 5A, B) while DNA transposons were more dispersed over the chromosomes, with some elements amplified and others excluded from the centromeric region (Fig. 5C–H).

The LTR_retriever analysis using the whole-genome data (Song *et al.*, 2018) also showed that ~50 % of the genome is represented by LTR retroelements (although with a large number of incomplete and unknown elements), supporting the RepeatExplorer graph-based clustering. In the repeat graphs (Fig. 3), LTR retroelements formed circular patterns (especially in the merged clusters based on overlapping reads) with a diverged domain of the LTRs including solo LTRs, where either the LTR has amplified and inserted independently, or from excision of the complete elements by illegitimate recombination, which is a frequent occurrence in plant genomes (Ma *et al.*, 2004; Jedlicka *et al.*, 2020). There seems to be high diversity in old retroelement families, and diverse elements in newly established, intact and lower copy-number element families. Some LTR retroelement RepeatExplorer clusters contained whole open reading frames (ORFs) and POL regions (such as CL35 and CL72) while others lacked the whole structures (such as CL19 and CL39). Compared with CL35 and CL72, CL21 had relatively more LTR copies, variants and divergent non-coding regions flanking the LTR in the graph, reflecting a greater proportion of excision events and perhaps greater age in the genome.

According to the degree of sequence diversity, most LTR retroelements have been inserted within the past few million years, and reflect a high rate of turnover (i.e. insertion and deletion) (Jedlicka *et al.*, 2020). Variation in TEs is widespread between closely related species and accessions, and, for example in *A. thaliana*, there is a wide TE variation in different ‘ecotypes’ (genotypes) and diverged insertions postdate (Ziolkowski *et al.*, 2009; Joly-Lopez and Bureau, 2014). In our study, the activity time of all LTR retroelements was mostly concentrated in 0.5–01 Mya (Fig. 1E), and a mass of diverged LTRs suggests that they suffered a rapid, massive gain of genomic content during evolutionary time.

There were several abundant ‘low-complexity’ clusters (examples in Fig. 6) with very few identified protein domains or homology to known repeats, but links to more defined clusters. ‘Low-complexity’ CL1 was linked to defined *Gypsy* clusters (CL26 and CL79) and ‘low-complexity’ CL2 was contained in a big circle linked to many clusters, where the two adjacent clusters were CL16 ‘simple repeat’ and CL67 ‘*Copia*’. CL4 was defined as *Copia*, which was connected to CL69 ‘low complexity’ and CL50 *Copia* (Supplementary Data Fig. S3 panels 2, 4, 6). These circular graphs depict gene domains and larger, more variable, domains with additional coverage and variability shown in the LTR region from intact and deleted (solo LTR) elements. FISH signal strength correlated and signal distribution along most chromosomes confirmed that these sequences are an important part of the *C. nankingense* genome. We suggest that most are related to degenerate and presumably ancient, very diverse solo LTRs and other parts of retroelements. This large proportion of fragments was not homologous to known sequences as such, perhaps being an unexplored aspect of sequence variation and amplification in many species that is revealed in *Chrysanthemum* and as a consequence has influence on generating diversity and on species evolution.

The complexity of the RepeatExplorer clustering graphs shows why sequences are a challenge for assemblies of whole genomes from high-accuracy short reads, while the variability is a challenge for long-read approaches with low accuracy. Apart from collapsing reads representing multiple repeats in the genome to a short site, contigs will often end with a repetitive sequence where alternative links can be made to multiple other contigs (Baker, 2012).

### Conclusions

As in most plant genomes, the majority of the 3000-Mb genome of *C. nankingense* is composed of highly repetitive DNA sequences, but an unusually low proportion of satellite tandem repeated DNA families. The characterization of repetitive sequences and their relationship to coding sequences is a necessary part of defining the pangenome of a genus, including structural variations in chromosomal sequences. Retrotransposons make up about half of the *C. nankingense* genome using reference-free assembly of DNA reads by either graph-based clustering or analysis of highly repetitive *k*-mer sequences. Some, mostly younger, retrotransposons showed little diversity and no excess of LTRs, while others have much higher copy numbers or diversity of LTRs, many as solo LTRs; in total, the analysis of both whole-genome assembly and short reads revealed that intact LTR retroelements represented <10 % of total LTR retroelement-related sequence. With the widespread genome distribution (shown by the *in situ* hybridization results), it is notable that there is no strong exclusion of retroelements or the ‘low-complexity’ elements with unknown homologies, but, as we speculate, likely derived from the LTRs of ancient retroelements from particular genome domains; this shows that the genome has the capacity to include and tolerate dispersed non-coding sequences.

The variation in rearranged and degenerate features of repeats we have identified in the *C. nankingense* genome shows how the genome landscape has been shaped during time through mutation, recombination and more element-specific processes such as transposition. Accumulation of repeats, and sometimes loss, along with chromosomal rearrangements occurs throughout evolutionary time. Degeneration of retroelement sequences leads to their silencing and inactivity, and, with recombination, may eliminate identifiable coding sequences, making TE family identification difficult (Mirouze and Vitte, 2014). However, the response of genomes to invasive elements may be more active through epigenetic mechanisms, including methylation or RNA silencing (e.g. Vicient and Casacuberta, 2017; Richert-Pöggeler *et al.*, 2021; Schmidt *et al.*, 2021) and influences genome features that may affect larger chromatin domains and the included genes.

As a consequence, retroelement mobility has an influence on the generation of diversity and species evolution, through silencing, and reactivation by stress, genomic fracturing or disease. The diploid *C. nankingense* is ancestral to polyploid taxa such as *C. indicum* (2*n* = 4*x* = 36), the main species used for horticultural flowers. In allopolyploids, turnover and differential homogenization of retroelements in the diploid ancestors may mean the silencing mechanisms have different effects on the two genomes coming together in the polyploid (Vicient and Casacuberta, 2017), and study of individual families, their ages and diversification in diploids, as here, may suggest ways multiple genomes interact.

## SUPPLEMENTARY DATA

Supplementary data are available online at https://academic.oup.com/aob and consists of the following: Figure S1: genomic organization of clusters CL110, CL113 and CL127. Figure S2: proportion of LTR retroelement lineages *Copia* and *Gypsy.*Figure S3: links between RepeatExplorer clusters. Figure S4: linked circular graphs of LTR retroelements. Table S1: RepeatExplorer sequence clustering results in *C. nankingense*. Table S2: *k*-mer assembly statistics. Table S3: primer sequences used in this study. Table S4: proportion of repetitive DNA sequences in the *C. nankingense* genome.

mcac066_suppl_Supplementary_Figure_S1

mcac066_suppl_Supplementary_Figure_S2

mcac066_suppl_Supplementary_Figure_S3

mcac066_suppl_Supplementary_Figure_S4

mcac066_suppl_Supplementary_Table_S1

mcac066_suppl_Supplementary_Table_S2

mcac066_suppl_Supplementary_Table_S3

mcac066_suppl_Supplementary_Table_S4

## References

[CIT0002] Baker M . 2012. De novo genome assembly: what every biologist should know. Nature Methods 9: 333–337. doi:10.1038/nmeth.1935.

[CIT0003] Belser C, Istace B, Denis E, et al. 2018. Chromosome-scale assemblies of plant genomes using nanopore long reads and optical maps. Nature Plants 4: 879–887. doi:10.1038/s41477-018-0289-4. 30390080

[CIT0004] Biscotti MA, Olmo E, Heslop-Harrison JS. 2015. Repetitive DNA in eukaryotic genomes. Chromosome Research 23: 415–420. doi:10.1007/s10577-015-9499-z. 26514350

[CIT0005] Bowen NJ, McDonald JF. 2001. *Drosophila* euchromatic LTR retrotransposons are much younger than the host species in which they reside. Genome Research 11: 1527–1540. doi:10.1101/gr.164201. 11544196 PMC311128

[CIT0006] Cheng ZJ, Murata M. 2003. A centromeric tandem repeat family originating from a part of Ty3/*gypsy*-retroelement in wheat and its relatives. Genetics 164: 665–672. doi:10.1093/genetics/164.2.665. 12807787 PMC1462596

[CIT0007] Fan M, Gao Y, Gao Y, Wu Z, Liu H, Zhang Q. 2019. Characterization and development of EST-SSR markers from transcriptome sequences of chrysanthemum (*Chrysanthemum* × *morifolium* Ramat.). HortScience 54: 772–778. doi:10.21273/hortsci13694-18.

[CIT0008] Flavell RB . 1982. Chromosomal DNA sequences and their organization. In: Parthier B, Boulter D. eds. Nucleic Acids and Proteins in Plants II. Encyclopedia of Plant Physiology, Vol. 14. Berlin, Heidelberg: Springer, 46–74. doi:10.1007/978-3-642-68347-3_2.

[CIT0009] Flavell AJ, Dunbar E, Anderson R, Pearce SR, Hartley R, Kumar A. 1992 *a*. Ty1-copia group retrotransposons are ubiquitous and heterogeneous in higher plants. Nucleic Acids Research 20: 3639–3644. doi:10.1093/nar/20.14.3639. 1379359 PMC334012

[CIT0010] Flavell AJ, Smith DB, Kumar A. 1992 *b*. Extreme heterogeneity of Ty1-copia group retrotransposons in plants. Molecular and General Genetics 231: 233–242. doi:10.1007/BF00279796. 1370976

[CIT0012] Frello S, Heslop-Harrison JS. 2000. Repetitive DNA sequences in *Crocus vernus* Hill (Iridaceae): the genomic organization and distribution of dispersed elements in the genus *Crocus* and its allies. Genome 43: 902–909. doi:10.1139/g00-044. 11081982

[CIT0013] Goffová I, Fajkus J. 2021. The rDNA loci-intersections of replication, transcription, and repair pathways. International Journal of Molecular Sciences 22: 302. doi:10.3390/ijms22031302. PMC7865372 33525595

[CIT0014] Gong Z, Wu Y, Koblížková A, et al 2012. Repeatless and repeat-based centromeres in potato: implications for centromere evolution. Plant Cell 24: 3559–3574. doi:10.1105/tpc.112.100511. 22968715 PMC3480287

[CIT0070] Hansen C, Heslop-Harrison JS. 2004. Sequences and phylogenies of plant pararetroviruses, viruses, and transposable elements. In: Advances in Botanical Research, Vol. 41. Academic Press, 165–193. doi:10.1016/S0065-2296(04)41004-0.

[CIT0015] He L, Liu J, Torres GA, Zhang H, Jiang J, Xie C. 2013. Interstitial telomeric repeats are enriched in the centromeres of chromosomes in *Solanum* species. Chromosome Research 21: 5–13. 23250588 10.1007/s10577-012-9332-x

[CIT0016] He Q, Cai Z, Hu T, et al 2015. Repetitive sequence analysis and karyotyping reveals centromere-associated DNA sequences in radish (*Raphanus sativus* L.). BMC Plant Biology 15: 1–12. doi:10.1007/s10577-012-9332-x. 25928652 PMC4417506

[CIT0017] Hemleben V, Kovarik A, Torres-Ruiz RA, Volkov RA, Thengiz Beridze T. Plant highly repeated satellite DNA: molecular evolution, distribution and use for identification of hybrids. Systematics and Biodiversity 5: 277–289. doi:10.1017/S147720000700240X.

[CIT0018] Heslop-Harrison JS, Schwarzacher T. 2011. Organisation of the plant genome in chromosomes. Plant Journal 66: 18–33. doi:10.1111/j.1365-313X.2011.04544.x. 21443620

[CIT0019] Heslop-Harrison JS, Schwarzacher T. 2013. Nucleosomes and centromeric DNA packaging. Proceedings of the National Academy of Sciences of the USA 110: 19974–19975. doi:10.1073/pnas.1319945110. 24282300 PMC3864337

[CIT0020] Jedlicka P, Lexa M, Kejnovsky E. 2020. What can long terminal repeats tell us about the age of LTR retrotransposons, gene conversion and ectopic recombination? Frontiers in Plant Science 11: 644. doi:10.3389/fpls.2020.00644. 32508870 PMC7251063

[CIT0021] Jesionek W, Bodláková M, Kubát Z, et al 2021. Fundamentally different repetitive element composition of sex chromosomes in *Rumex acetosa*. Annals of Botany 127: 33–47. doi:10.1093/aob/mcaa160. 32902599 PMC7750719

[CIT0022] Jiang J, Gill BS. 2006. Current status and the future of fluorescence in situ hybridization (FISH) in plant genome research. Genome 49: 1057–1068. doi:10.1139/g06-076. 17110986

[CIT0023] Joly-Lopez Z, Bureau TE. 2014. Diversity and evolution of transposable elements in *Arabidopsis*. Chromosome Research 22: 203–216. doi:10.1007/s10577-014-9418-8. 24801342

[CIT0024] Kimura M, Ohta T. 1972. On the stochastic model for estimation of mutational distance between homologous proteins. Journal of Molecular Evolution 2: 87–90. doi:10.1007/BF01653945. 4668865

[CIT0025] Lin Y, Yuan J, Kolmogorov M, Shen MW, Chaisson M, Pevzner PA. 2016. Assembly of long error-prone reads using de Bruijn graphs. Proceedings of the National Academy of Sciences of the USA 113: E8396–E8405. doi:10.1073/pnas.1604560113. 27956617 PMC5206522

[CIT0026] Liu Q, Li X, Zhou X, et al 2019. The repetitive DNA landscape in *Avena* (Poaceae): chromosome and genome evolution defined by major repeat classes in whole-genome sequence reads. BMC Plant Biology 19: 1–17. doi:10.1186/s12870-019-1769-z. 31146681 PMC6543597

[CIT0027] Ma J, Jackson SA. 2006. Retrotransposon accumulation and satellite amplification mediated by segmental duplication facilitate centromere expansion in rice. Genome Research 16: 251–259. doi:10.1101/gr.4583106. 16354755 PMC1361721

[CIT0028] Ma J, Devos KM, Bennetzen JL. 2004. Analyses of LTR-retrotransposon structures reveal recent and rapid genomic DNA loss in rice. Genome Research 14: 860–869. doi:10.1101/gr.1466204. 15078861 PMC479113

[CIT0029] Ma YP, Chen MM, Wei JX, et al 2016. Origin of *Chrysanthemum* cultivars – evidence from nuclear low-copy *LFY* gene sequences. Biochemical Systematics and Ecology 65: 129–136. doi:10.1016/j.bse.2016.02.010.

[CIT0030] Ma YP, Zhao L, Zhang WJ, et al 2020. Origins of cultivars of *Chrysanthemum* – evidence from the chloroplast genome and nuclear *LFY* gene. Journal of Systematics and Evolution 58: 925–944. doi:10.1111/jse.12682.

[CIT0031] Macas J, Novák P, Pellicer J, et al 2015. In depth characterization of repetitive DNA in 23 plant genomes reveals sources of genome size variation in the legume tribe Fabeae. PLoS One 10: e0143424. doi:10.1371/journal.pone.0143424. 26606051 PMC4659654

[CIT0032] Marcais G, Kingsford C. 2011. A fast, lock-free approach for efficient parallel counting of occurrences of k-mers. Bioinformatics 27: 764–770. doi:10.1093/bioinformatics/btr011. 21217122 PMC3051319

[CIT0033] Mehrotra S, Goyal V. 2014. Repetitive sequences in plant nuclear DNA: types, distribution, evolution and function. Genomics Proteomics & *Bioinformatics* 12: 164–171. doi:10.1016/j.gpb.2014.07.003. PMC4411372 25132181

[CIT0034] Mirouze M, Vitte C. 2014. Transposable elements, a treasure trove to decipher epigenetic variation: insights from *Arabidopsis* and crop epigenomes. Journal of Experimental Botany 65: 2801–2812. doi:10.1093/jxb/eru120. 24744427

[CIT0035] Negm S, Greenberg A, Larracuente AM, Sproul JS. 2021. RepeatProfiler: a pipeline for visualization and comparative analysis of repetitive DNA profiles. Molecular Ecology Resources 21: 969–981. doi:10.1111/1755-0998.13305. 33277787 PMC7954937

[CIT0072] Neil O, Anderson. 2006. Chrysanthemum. In: Flower Breeding and Genetics: Issues, Challenges and Opportunities for the 21st Century. New York: Springer, 389–437.

[CIT0036] Neumann P, Novák P, Hoštáková N, Macas J. 2019. Systematic survey of plant LTR-retrotransposons elucidates phylogenetic relationships of their polyprotein domains and provides a reference for element classification. Mobile DNA 10: 1–17. doi:10.1186/s13100-018-0144-1. 30622655 PMC6317226

[CIT0037] Niu XM, Xu YC, Li ZW, et al 2019. Transposable elements drive rapid phenotypic variation in *Capsella rubella*. Proceedings of the National Academy of Sciences of the USA 116: 6908–6913. doi:10.1073/pnas.1811498116. 30877258 PMC6452725

[CIT0038] Novák P, Neumann P, Macas J. 2010. Graph-based clustering and characterization of repetitive sequences in next-generation sequencing data. BMC Bioinformatics 11: 378. doi:10.1186/1471-2105-11-378. 20633259 PMC2912890

[CIT0039] Novák P, Neumann P, Pech J, Steinhaisl J, Macas J. 2013. RepeatExplorer: a Galaxy-based web server for genome-wide characterization of eukaryotic repetitive elements from next generation sequence reads. Bioinformatics 29: 792–793. doi:10.1093/bioinformatics/btt054. 23376349

[CIT0040] Novák P, Neumann P, Macas J. 2020. Global analysis of repetitive DNA from unassembled sequence reads using RepeatExplorer2. Nature Protocols 15: 3745–3776. doi:10.1038/s41596-020-0400-y. 33097925

[CIT0041] Ou S, Jiang N. 2018. LTR_retriever: a highly accurate and sensitive program for identification of long terminal repeat retrotransposons. Plant Physiology 176: 1410–1422. doi:10.1104/pp.17.01310. 29233850 PMC5813529

[CIT0042] Pereira V . 2004. Insertion bias and purifying selection of retrotransposons in the *Arabidopsis thaliana* genome. Genome Biology 5: R79. doi:10.1186/gb-2004-5-10-r79. 15461797 PMC545599

[CIT0043] Porebski S, Bailey LG, Baum BR. 1997. Modification of a CTAB DNA extraction protocol for plants containing high polysaccharide and polyphenol components. Plant Molecular Biology Reporter 15: 8–15. doi:10.1007/BF02772108.

[CIT0044] Quesneville H . 2020. Twenty years of transposable element analysis in the *Arabidopsis thaliana* genome. Mobile DNA 11: 1–13. doi:10.1186/s13100-020-00223-x. 32742313 PMC7385966

[CIT0045] Rao SR, Trivedi S, Emmanuel D, Merita K, Hynniewta M. 2010. DNA repetitive sequences-types, distribution and function: a review. Journal of Cell and Molecular Biology 7: 1–11.

[CIT0046] Richert-Pöggeler KR, Vijverberg K, Alisawi O, Chofong GN, Schwarzacher T, Heslop-Harrison JS. 2021. Participation of multifunctional RNA in replication, recombination and regulation of endogenous plant pararetroviruses (EPRVs). Frontiers in Plant Science 12: 1148. doi:10.3389/fpls.2021.689307. PMC8256270 34234799

[CIT0047] Schmidt T . 1999. LINEs, SINEs and repetitive DNA: non-LTR retrotransposons in plant genomes. Plant Molecular Biology 40: 903–910. doi: 10.1023/A:1006212929794. 10527415

[CIT0048] Schmidt N, Seibt KM, Weber B, Schwarzacher T, Schmidt T, Heitkam T. 2021. Broken, silent, and in hiding: tamed endogenous pararetroviruses escape elimination from the genome of sugar beet (*Beta vulgaris*). Annals of Botany 128: 281–299. doi:10.1093/aob/mcab042. 33729490 PMC8389469

[CIT0071] Schrader L, Schmitz J. 2019. The impact of transposable elements in adaptive evolution. Molecular Ecology 28: 1537–1549. doi:10.1111/mec.14794. 30003608

[CIT0049] Schulman AH . 2013. Retrotransposon replication in plants. Current Opinion in Virology 3: 604–614. doi:10.1016/j.coviro.2013.08.009. 24035277

[CIT0050] Schwarzacher T . 2016. Preparation and fluorescent analysis of plant metaphase chromosomes. In: Caillaud MC. ed. Plant Cell Division, Methods in Molecular Biology, Vol. 1370. New York, NY: Humana Press, 87–103. doi:10.1007/978-1-4939-3142-2_7. 26659956

[CIT0051] Schwarzacher T, Heslop-Harrison JS. 2000. Practical in situ hybridization. Oxford, UK: BIOS Scientific Publishers Ltd.

[CIT0052] Shahrajabian MH, Sun W, Zandi P, Cheng Q. 2019. A review of *Chrysanthemum*, the eastern queen in traditional Chinese medicine with healing power in modern pharmaceutical sciences. Applied Ecology and Environmental Research 17: 13355–13369. doi:10.15666/aeer/1706_1335513369.

[CIT0053] Sharma A, Wolfgruber TK, Presting GG. 2013. Tandem repeats derived from centromeric retrotransposons. BMC Genomics 14: 142. doi:10.1186/1471-2164-14-142. 23452340 PMC3648361

[CIT0054] Sindi SS, Hunt BR, Yorke JA. 2008. Duplication count distributions in DNA sequences. Physical Review E 78: 061912. doi:10.1103/PhysRevE.78.061912. PMC3121164 19256873

[CIT0055] Song C, Liu Y, Song A, et al 2018. The *Chrysanthemum nankingense* genome provides insights into the evolution and diversification of chrysanthemum flowers and medicinal traits. Molecular Plant 11: 1482–1491. doi:10.1016/j.molp.2018.10.003. 30342096

[CIT0056] Stritt C, Wyler M, Gimmi EL, Pippel M, Roulin AC. 2020. Diversity, dynamics and effects of long terminal repeat retrotransposons in the model grass *Brachypodium distachyon*. New Phytologist 227: 1736–1748. doi:10.1111/nph.16308. 31677277 PMC7497039

[CIT0057] Stuart T, Eichten SR, Cahn J, Karpievitch YV, Borevitz JO, Lister R. 2016. Population scale mapping of transposable element diversity reveals links to gene regulation and epigenomic variation. eLife 5: e20777. doi:10.7554/eLife.20777. 27911260 PMC5167521

[CIT0058] Vershinin AV, Heslop-Harrison JS. 1998. Comparative analysis of the nucleosomal structure of rye, wheat and their relatives. Plant Molecular Biology 36: 149–161. doi:10.1023/A:1005912822671. 9484470

[CIT0059] Vershinin AV, Druka A, Alkhimova AG, Kleinhofs A, Heslop-Harrison JS. 2002. LINEs and *gypsy*-*like* retrotransposons in *Hordeum* species. Plant Molecular Biology 49: 1–14. doi:10.1023/A:1014469830680. 12008894

[CIT0060] Vicient CM, Casacuberta JM. 2017. Impact of transposable elements on polyploid plant genomes. Annals of Botany 120: 195–207. doi:10.1093/aob/mcx078. 28854566 PMC5737689

[CIT0061] Vitales D, Garcia S, Dodsworth S. 2020. Reconstructing phylogenetic relationships based on repeat sequence similarities. Molecular Phylogenetics and Evolution 147: 106766. doi:10.1016/j.ympev.2020.106766. 32119996

[CIT0062] Wang ZW, Rouard M, Biswas M, et al. 2021. A chromosome-level reference genome of *Ensete glaucum* gives insight into diversity, chromosomal and repetitive sequence evolution in the Musaceae. GigaScience 11: giac027. doi:10.1093/gigascience/giac027. PMC9055855 35488861

[CIT0063] Wang J, Xiao H. 2013. Discrimination of different white chrysanthemum by electronic tongue. Journal of Food Science and Technology 50: 986–992. doi:10.1007/s13197-011-0422-0. 24426007 PMC3722412

[CIT0064] Wendel JF, Jackson SA, Meyers BC, Wing RA. 2016. Evolution of plant genome architecture. Genome Biology 17: 1–14. doi:10.1186/s13059-016-0908-1. 26926526 PMC4772531

[CIT0065] Wright SI, Agrawal N. Bureau TE. 2003. Effects of recombination rate and gene density on transposable element distributions in *Arabidopsis thaliana*. Genome Research 13: 1897–1903. doi:10.1101/gr.1281503. 12902382 PMC403781

[CIT0066] Xiong W, He L, Lai J, Dooner HK, Du C. 2014. HelitronScanner uncovers a large overlooked cache of Helitron transposons in many plant genomes. Proceedings of the National Academy of Sciences of the USA 111: 10263–10268. doi:10.1073/pnas.1410068111. 24982153 PMC4104883

[CIT0067] Xiong W, Dooner HK, Du C. 2016. Rolling-circle amplification of centromeric Helitrons in plant genomes. Plant Journal 88: 1038–1045. doi:10.1111/tpj.13314. 27553634

[CIT0068] Zhang RG, Wang ZX, Ou S, Li GY. 2019. TEsorter: lineage-level classification of transposable elements using conserved protein domains. bioRxiv: 800177. doi:10.1101/800177.

[CIT0069] Ziolkowski PA, Koczyk G, Galganski L, Sadowski J. 2009. Genome sequence comparison of Col and Ler lines reveals the dynamic nature of *Arabidopsis* chromosomes. Nucleic Acids Research 37: 3189–3201. doi:10.1093/nar/gkp183. 19305000 PMC2691826

