# Supplementary material for: The nature and genomic landscape of repetitive DNA classes in Chrysanthemum nankingense shows recent genomic changes
Source: Ann Bot. 2022 May 27;131(1):215–28. doi: 10.1093/aob/mcac066 (PMC9904347; doi:10.1093/aob/mcac066)
Supplement: mcac066_suppl_Supplementary_Figure_S1 [file mcac066_suppl_supplementary_figure_s1.docx]

Zhang et al. The nature and genomic landscape of repetitive DNA classes in *Chrysanthemum nankingense* shows recent genomic changes


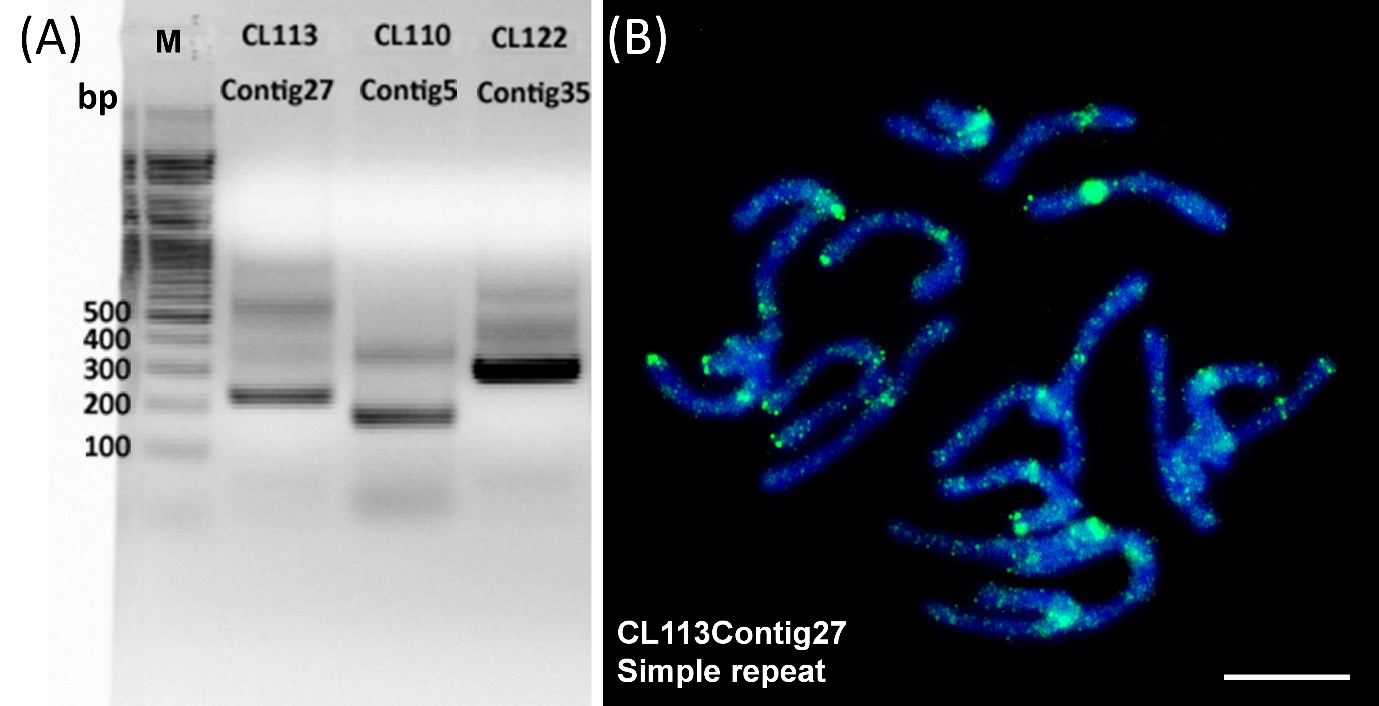


**Fig. S1 Genomic organisation of Clusters CL110, CL113 and CL127.**

(A) PCR of fragments of CL113Contig27, CL110Contig5 and CL122Contig35 using primers as described in Supplementary Table S3.

(B) Fluorescent *in situ* hybridization of CL113Contig27, the fluorescence signals of CL110Contig5 and CL122Contig35 are shown in Fig. 2E and Fig. 5B, respectively. Bar = 10 μm.
