# Supplementary material for: The nature and genomic landscape of repetitive DNA classes in Chrysanthemum nankingense shows recent genomic changes
Source: Ann Bot. 2022 May 27;131(1):215–28. doi: 10.1093/aob/mcac066 (PMC9904347; doi:10.1093/aob/mcac066)
Supplement: mcac066_suppl_Supplementary_Figure_S2 [file mcac066_suppl_supplementary_figure_s2.docx]

Zhang et al. The nature and genomic landscape of repetitive DNA classes in *Chrysanthemum nankingense* shows recent genomic changes


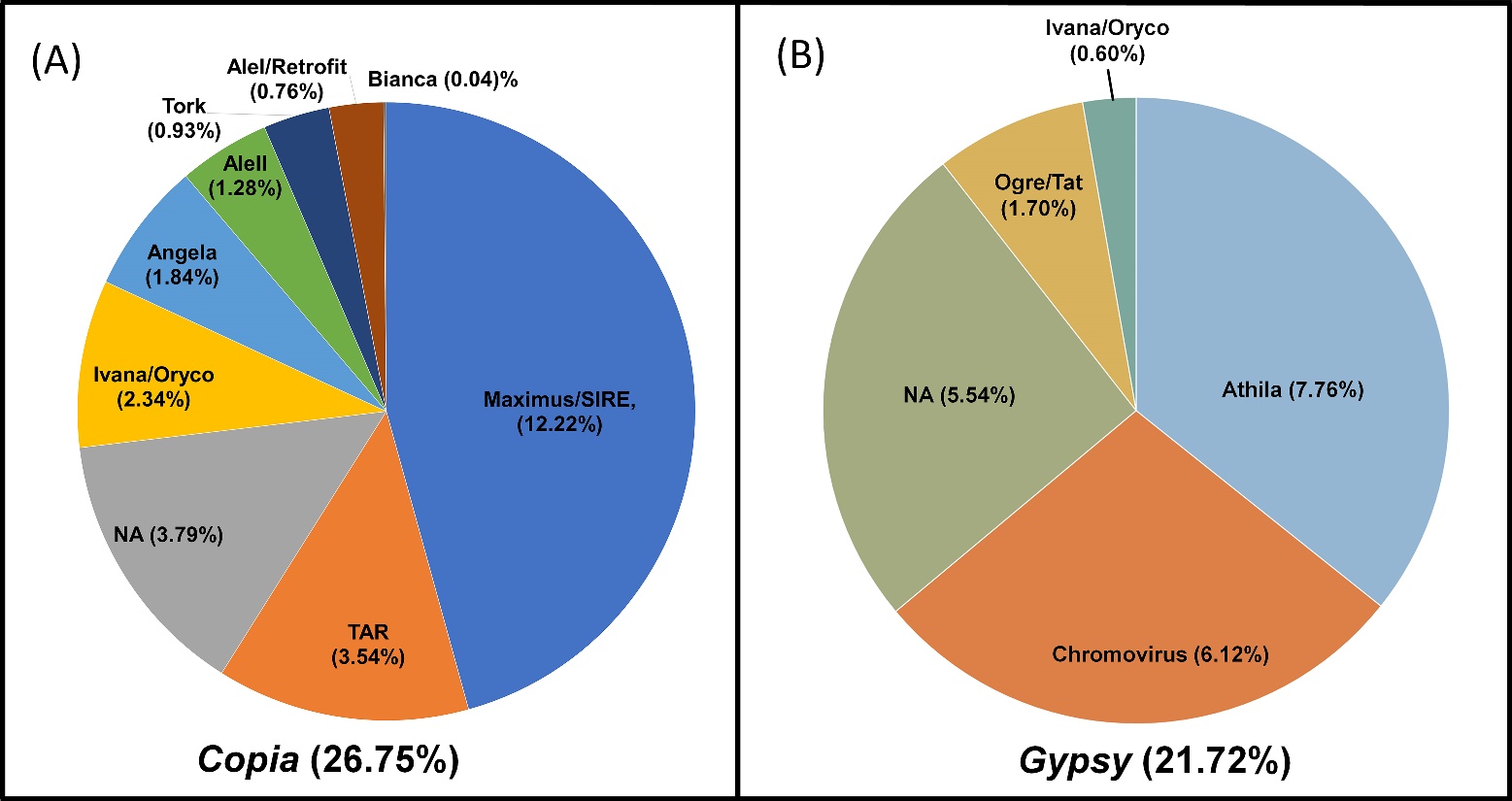


**Fig. S2 Proportion of LTR-retroelement lineages (A) *Copia* and (B) *Gypsy*.**

LTR-retroelement sequences were classified into distinct lineages based on differences in structural and sequence features of the elements relying three most conserved polyprotein domains, RT, RH and INT (Neumann *et al.*, 2019). For individual classification see Supplementary Table S1. NA: unclassified group
