# Supplementary material for: The nature and genomic landscape of repetitive DNA classes in Chrysanthemum nankingense shows recent genomic changes
Source: Ann Bot. 2022 May 27;131(1):215–28. doi: 10.1093/aob/mcac066 (PMC9904347; doi:10.1093/aob/mcac066)
Supplement: mcac066_suppl_Supplementary_Figure_S3 [file mcac066_suppl_supplementary_figure_s3.docx]

Zhang et al. The nature and genomic landscape of repetitive DNA classes in *Chrysanthemum nankingense* shows recent genomic changes


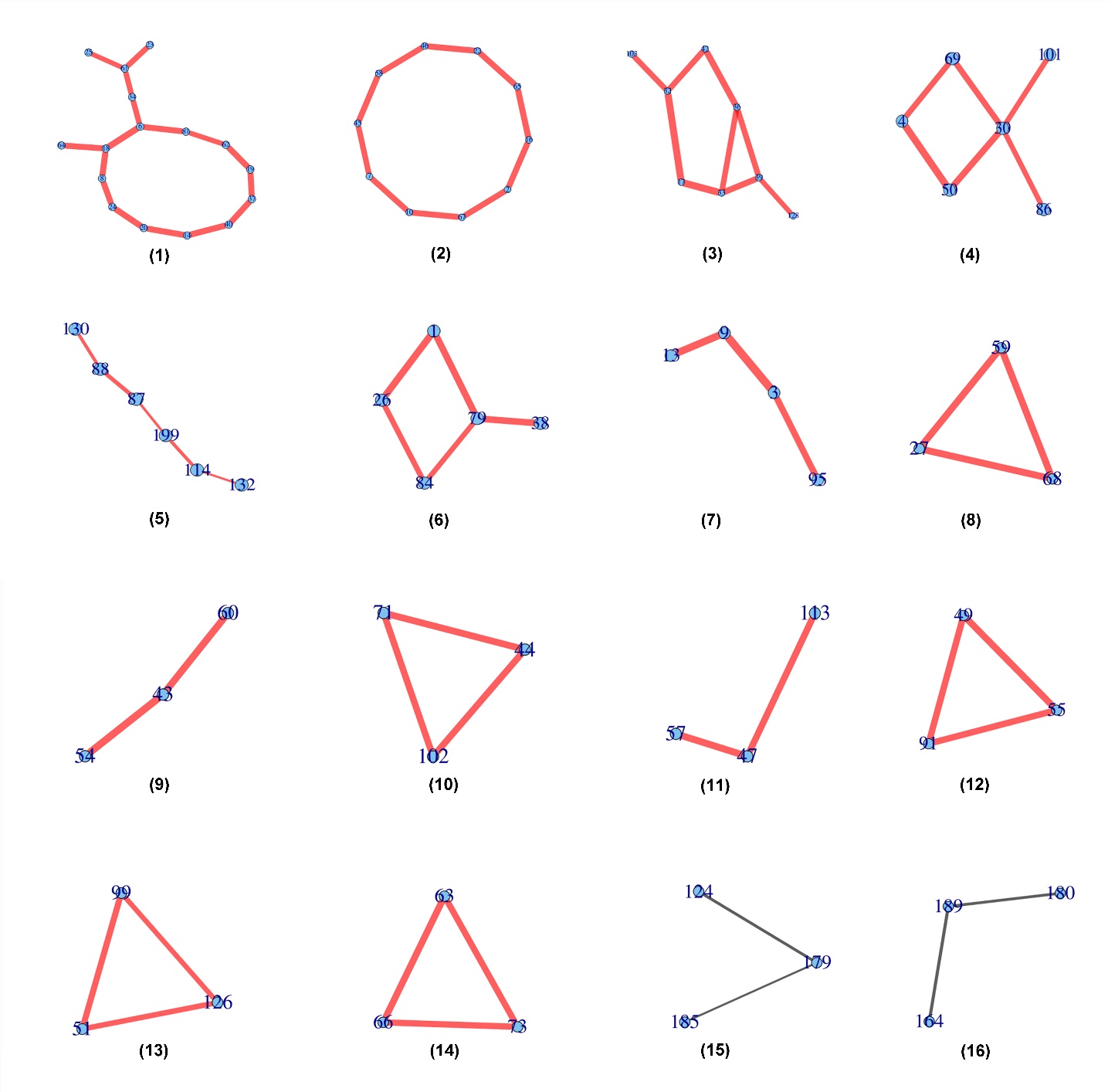


**Fig. S3 Links between RepeatExplorer Clusters in *Chrysanthemum nankingense.***

Sixteen groups linking more than two clusters were obtained with the cutoff set 0.1. Connection through mates is labelled by red (panels 1-14) if similarity hits exist between clusters, otherwise connection is shown as grey (panels 15, 16).
