# Supplementary material for: The nature and genomic landscape of repetitive DNA classes in Chrysanthemum nankingense shows recent genomic changes
Source: Ann Bot. 2022 May 27;131(1):215–28. doi: 10.1093/aob/mcac066 (PMC9904347; doi:10.1093/aob/mcac066)
Supplement: mcac066_suppl_Supplementary_Figure_S4 [file mcac066_suppl_supplementary_figure_s4.docx]

Zhang et al. The nature and genomic landscape of repetitive DNA classes in *Chrysanthemum nankingense* shows recent genomic changes


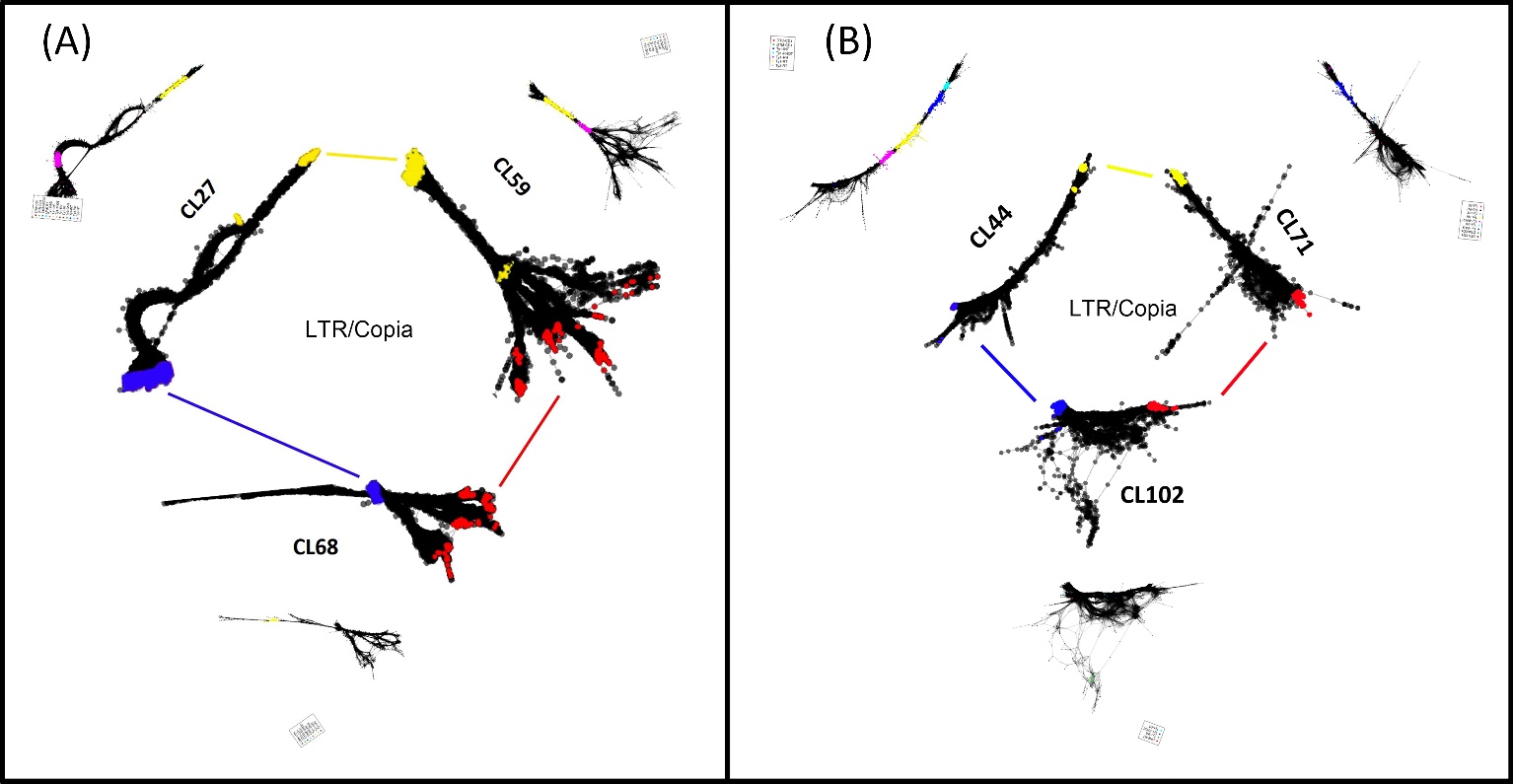


**Fig. S4 Linked circular graphs of LTR-retroelements.**

The painted regions at the end of each cluster with red, yellow and blue represented the reads with similarity to all other neighbouring clusters, which were connected by the same-coloured lines in the graphs.

(A) Connections between CL27, CL59 and CL68. In the small graphs next to each cluster, the CL27 showed the GAG (red rose), PROT (grey) and INT (yellow) domains, CL59 showed the RT (yellow) and RH (red rose) domains, CL68 showed a little GAG (yellow) domain.

(B) Connections between CL44, CL71 and CL102. CL44 showed the POL (PROT-INT-RT-RH) (blue green-blue-yellow-red rose), links to CL71 (GAG, blue) and CL102 (LTR, black).
