# Supplementary material for: The nature and genomic landscape of repetitive DNA classes in Chrysanthemum nankingense shows recent genomic changes
Source: Ann Bot. 2022 May 27;131(1):215–28. doi: 10.1093/aob/mcac066 (PMC9904347; doi:10.1093/aob/mcac066)
Supplement: mcac066_suppl_Supplementary_Table_S1 [file mcac066_suppl_supplementary_table_s1.docx]

Zhang et al. The nature and genomic landscape of repetitive DNA classes in *Chrysanthemum nankingense* shows recent genomic changes

Supplementary Table S1: RepeatExplorer cluster results in *Chrysanthemum nankingense.*

266 clusters have at least 0.01% genome proportion (GP) and make up 69% of the genome. Repeat classes were automatically identified using Repeat Masker and the number and % of hits among the number of reads in the cluster are given; those in bold are > 3%. The retroelement domain hits were used to determine the retroelement lineage (see Figure S1). The clusters used for FISH were highlighted yellow and showed the layout graphs.

| **Cluster** | **Total length (bp)** | **Number of reads** | **Genome proportion (GP) (%)** | **Cumulative GP (%)** | **Repeat Masker** | **Retroelement Domain hits** | **Retroelement Lineage** | **Layout** |
| --- | --- | --- | --- | --- | --- | --- | --- | --- |
| CL1 | 7495250 | 29981 | 1.4 | 1.4 | Low_complexity (**5143hits, 4.47%**) | Ty3-GAG Ty3/gypsy Athila (2450 hits 8.17%) | Athila | 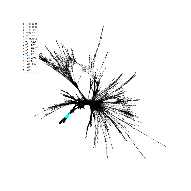 |
| CL2 | 7155250 | 28621 | 1.34 | 2.7 | Low_complexity (131hits, 0.127%) | Ty1-RT Ty1/copia Maximus/SIRE (15 hits 0.0524%) | Maximus/SIRE | 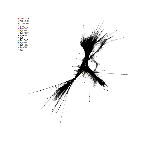 |
| CL3 | 7109500 | 28438 | 1.33 | 4.1 | LTR.Gypsy (**4313hits, 3.99%**) | DTA-CD1 NA NA (43 hits 0.151%) | NA |  |
| CL4 | 7057750 | 28231 | 1.32 | 5.4 | LTR.Copia (864hits, 0.998%) | Ty1-RT Ty1/copia TAR (17 hits 0.0602%) | TAR | 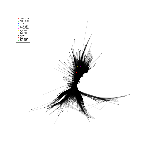 |
| CL5 | 6883500 | 27534 | 1.29 | 6.7 | LTR.Gypsy (600hits, 0.674%) | Ty3-GAG Ty3/gypsy chromovirus (73 hits 0.265%) | chromovirus |  |
| CL6 | 6798750 | 27195 | 1.27 | 8 | LTR.Copia (1867hits, 1.2%) | Ty1-RH Ty1/copia TAR (4 hits 0.0147%) | TAR | 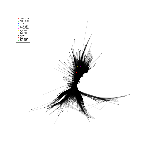 |
| CL7 | 5822250 | 23289 | 1.09 | 9.1 | Simple_repeat (69hits, 0.182%) | DHH-CD1 NA NA (13 hits 0.0558%) | NA | 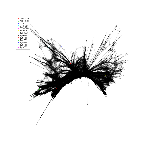 |
| CL8 | 5655250 | 22621 | 1.06 | 10.1 | LTR.Gypsy (473hits, 0.304%) | DTM-CD1 NA NA (35 hits 0.155%) | NA |  |
| CL9 | 5634250 | 22537 | 1.06 | 11.2 | Simple_repeat (400hits, 0.417%) | Ty3-GAG Ty3/gypsy chromovirus (199 hits 0.883%) | chromovirus | 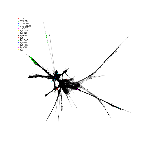 |
| CL10 | 5547250 | 22189 | 1.04 | 12.2 | Low_complexity (21hits, 0.0369%) | LINE-ENDO NA NA (21 hits 0.0946%) | NA | 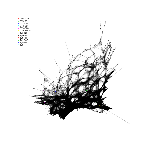 |
| CL11 | 5538250 | 22153 | 1.04 | 13.2 | LTR.Gypsy (671hits, 0.699%) | Ty3-GAG Ty3/gypsy Athila (896 hits 4.04%) | Athila |  |
| CL12 | 5246250 | 20985 | 0.982 | 14.2 | LTR.Copia (340hits, 0.923%) | Ty1-GAG Ty1/copia Angela (566 hits 2.7%) | Angela |  |
| CL13 | 5139750 | 20559 | 0.962 | 15.2 | LTR.Gypsy (**3790hits, 14.6%**) | Ty3-INT Ty3/gypsy chromovirus (1243 hits 6.05%) | chromovirus |  |
| CL14 | 5100000 | 20400 | 0.955 | 16.1 | LTR.Copia (13hits, 0.0182%) | Ty1-INT Ty1/copia AleII (7 hits 0.0343%) | AleII |  |
| CL15 | 4930250 | 19721 | 0.923 | 17.1 | LTR.Caulimovirus (**3055hits, 4.27%**) | Ty1-RT Ty1/copia Maximus/SIRE (17 hits 0.0862%) | Maximus/SIRE |  |
| CL16 | 4874500 | 19498 | 0.913 | 18 | Simple_repeat (**2293hits, 3.91%**) | Ty1-GAG Ty1/copia Ivana/Oryco (1093 hits 5.61%) | Ivana/Oryco |  |
| CL17 | 4640000 | 18560 | 0.869 | 18.8 | LTR.Gypsy (37hits, 0.0345%) | DTA-CD1 NA NA (28 hits 0.151%) | NA |  |
| CL18 | 4632500 | 18530 | 0.868 | 19.7 | LTR.Copia (6hits, 0.00814%) | Ty1-GAG Ty1/copia Tork (26 hits 0.14%) | Tork |  |
| CL19 | 4534750 | 18139 | 0.849 | 20.6 | LTR.Copia (**12120hits, 57.8%**) | Ty1-RT Ty1/copia Maximus/SIRE (10198 hits 56.2%) | Maximus/SIRE |  |
| CL20 | 4499750 | 17999 | 0.843 | 21.4 | LTR.Copia (259hits, 0.355%) | LINE-RT NA NA (7 hits 0.0389%) | NA |  |
| CL21 | 4462250 | 17849 | 0.836 | 22.2 | LTR.Gypsy (**3806hits, 14%**) | Ty3-INT Ty3/gypsy Ogre/Tat (1826 hits 10.2%) | Ogre/Tat |  |
| CL22 | 4449000 | 17796 | 0.833 | 23.1 | RC.Helitron (43hits, 0.0777%) | Ty1-INT Ty1/copia Angela (10 hits 0.0562%) | Angela |  |
| CL23 | 4319250 | 17277 | 0.809 | 23.9 | LTR.Copia (57hits, 0.058%) | DTC-CD1 NA NA (7 hits 0.0405%) | NA |  |
| CL24 | 4223250 | 16893 | 0.791 | 24.7 | LTR.Copia (46hits, 0.0715%) | Ty1-GAG Ty1/copia Ivana/Oryco (3749 hits 22.2%) | Ivana/Oryco |  |
| CL25 | 4185500 | 16742 | 0.784 | 25.5 | LTR.Gypsy (531hits, 0.412%) | Ty3-RT Ty3/gypsy chromovirus (1 hits 0.006%) | chromovirus |  |
| CL26 | 4175250 | 16701 | 0.782 | 26.2 | LTR.Gypsy (266hits, 0.444%) | Ty3-GAG Ty3/gypsy Athila (205 hits 1.23%) | Athila |  |
| CL27 | 4170750 | 16683 | 0.781 | 27 | LTR.Copia (**4038hits, 12.5%**) | Ty1-INT Ty1/copia Maximus/SIRE (1718 hits 10.3%) | Maximus/SIRE |  |
| CL28 | 4139000 | 16556 | 0.775 | 27.8 | LTR.Gypsy (219hits, 0.64%) | Ty3-INT Ty3/gypsy chromovirus (137 hits 0.827%) | chromovirus |  |
| CL29 | 4079000 | 16316 | 0.764 | 28.6 | LTR.Copia (**2793hits, 11.4%**) | Ty1-RT Ty1/copia Maximus/SIRE (643 hits 3.94%) | Maximus/SIRE |  |
| CL30 | 4068000 | 16272 | 0.762 | 29.3 | Low_complexity (25hits, 0.0162%) | Ty1-GAG Ty1/copia Ivana/Oryco (40 hits 0.246%) | Ivana/Oryco |  |
| CL31 | 3988750 | 15955 | 0.747 | 30.1 | LTR.Copia (**3784hits, 17.5%**) | Ty1-RT Ty1/copia Maximus/SIRE (1085 hits 6.8%) | Maximus/SIRE |  |
| CL32 | 3954500 | 15818 | 0.741 | 30.8 | LTR.Gypsy (**3041hits, 4.74%**) | Ty3-GAG Ty3/gypsy Athila (15 hits 0.0945%) | Athila |  |
| CL33 | 3905250 | 15621 | 0.731 | 31.5 | LTR.Gypsy (1988hits, 1.68%) | Ty3-INT Ty3/gypsy Athila (11 hits 0.0704%) | Athila |  |
| CL34 | 3820000 | 15280 | 0.715 | 32.3 | Low_complexity (637hits, 1.04%) | DTM-CD1 NA NA (17 hits 0.111%) | NA |  |
| CL35 | 3770750 | 15083 | 0.706 | 33 | LTR.Copia (**10627hits, 61.9%**) | Ty1-RT Ty1/copia TAR (2274 hits 15.1%) | TAR | 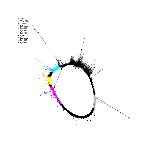 |
| CL36 | 3767250 | 15069 | 0.705 | 33.7 | LTR.Gypsy (**2895hits, 7.22%**) | Ty3-INT Ty3/gypsy Athila (99 hits 0.657%) | Athila |  |
| CL38 | 3719500 | 14878 | 0.697 | 35.1 | LTR.Gypsy (**13433hits, 81%**) | Ty3-INT Ty3/gypsy Athila (4806 hits 32.3%) | Athila |  |
| CL37 | 3722500 | 14890 | 0.697 | 34.4 | LTR.Copia (**11887hits, 57.9%**) | Ty1-INT Ty1/copia Maximus/SIRE (8761 hits 58.8%) | Maximus/SIRE |  |
| CL39 | 3629750 | 14519 | 0.68 | 35.7 | LTR.Gypsy (**1964hits, 8.71%**) | Ty3-GAG Ty3/gypsy Athila (3692 hits 25.4%) | Athila |  |
| CL40 | 3505000 | 14020 | 0.656 | 36.4 | LTR.Copia (**2800hits, 4.37%**) | Ty1-PROT Ty1/copia Ivana/Oryco (2140 hits 15.3%) | Ivana/Oryco |  |
| CL41 | 3474750 | 13899 | 0.651 | 37 | LTR.Copia (636hits, 0.886%) | DHH-CD1 NA NA (10 hits 0.0719%) | NA |  |
| CL42 | 3472250 | 13889 | 0.65 | 37.7 | LTR.Gypsy (**2723hits, 5.19%**) | LINE-RT NA NA (1 hits 0.0072%) | NA |  |
| CL43 | 3411000 | 13644 | 0.639 | 38.3 | LTR.Copia (**6474hits, 37.3%**) | Ty1-RT Ty1/copia Maximus/SIRE (2292 hits 16.8%) | Maximus/SIRE |  |
| CL44 | 3270500 | 13082 | 0.612 | 38.9 | LTR.Copia (**6147hits, 39.1%**) | Ty1-RT Ty1/copia Maximus/SIRE (2072 hits 15.8%) | Maximus/SIRE |  |
| CL45 | 3260250 | 13041 | 0.611 | 39.6 | LTR.Copia (**9555hits, 59.2%**) | Ty1-RH Ty1/copia Maximus/SIRE (4062 hits 31.1%) | Maximus/SIRE |  |
| CL46 | 3238250 | 12953 | 0.606 | 40.2 | LTR.Copia (**6783hits, 35%**) | Ty1-INT Ty1/copia Maximus/SIRE (4406 hits 34%) | Maximus/SIRE |  |
| CL47 | 3097500 | 12390 | 0.58 | 40.7 | LTR.Gypsy (157hits, 0.178%) | Ty1-GAG Ty1/copia Ivana/Oryco (490 hits 3.95%) | Ivana/Oryco |  |
| CL48 | 3077250 | 12309 | 0.576 | 41.3 | LTR.Gypsy (**1486hits, 7.57%**) | Ty3-GAG Ty3/gypsy Athila (616 hits 5%) | Athila |  |
| CL49 | 3002500 | 12010 | 0.562 | 41.9 | LTR.Copia (**1093hits, 3.01%**) | Ty1-GAG Ty1/copia Maximus/SIRE (1290 hits 10.7%) | Maximus/SIRE |  |
| CL50 | 2977250 | 11909 | 0.558 | 42.4 | LTR.Copia (1366hits, 2.51%) | LINE-RT NA NA (21 hits 0.176%) | NA |  |
| CL51 | 2910000 | 11640 | 0.545 | 43 | rRNA (**6444hits, 48%**) | Ty3-INT Ty3/gypsy chromovirus (9 hits 0.0773%) | chromovirus |  |
| CL52 | 2908750 | 11635 | 0.545 | 43.5 | LTR.Copia (**5753hits, 42%**) | Ty1-RT Ty1/copia Angela (1928 hits 16.6%) | Angela |  |
| CL53 | 2888250 | 11553 | 0.541 | 44.1 | Simple_repeat (84hits, 0.105%) | DHH-CD1 NA NA (1 hits 0.00866%) | NA |  |
| CL54 | 2848250 | 11393 | 0.533 | 44.6 | LTR.Copia (619hits, 1.42%) | Ty1-GAG Ty1/copia Ivana/Oryco (1098 hits 9.64%) | Ivana/Oryco |  |
| CL55 | 2818250 | 11273 | 0.528 | 45.1 | LTR.Copia (**8557hits, 64.1%**) | Ty1-RT Ty1/copia Maximus/SIRE (2871 hits 25.5%) | Maximus/SIRE |  |
| CL56 | 2760250 | 11041 | 0.517 | 45.6 | LTR.Gypsy (**2990hits, 21.3%**) | Ty3-INT Ty3/gypsy Athila (747 hits 6.77%) | Athila |  |
| CL57 | 2694750 | 10779 | 0.505 | 46.2 | LTR.Copia (**4911hits, 37.5%**) | Ty1-RT Ty1/copia Maximus/SIRE (1966 hits 18.2%) | Maximus/SIRE |  |
| CL58 | 2638500 | 10554 | 0.494 | 46.6 | LTR.Copia (**5707hits, 45.9%**) | Ty1-RT Ty1/copia Maximus/SIRE (5350 hits 50.7%) | Maximus/SIRE |  |
| CL59 | 2627750 | 10511 | 0.492 | 47.1 | LTR.Copia (**4699hits, 37.9%**) | Ty1-RT Ty1/copia Maximus/SIRE (2288 hits 21.8%) | Maximus/SIRE |  |
| CL60 | 2605250 | 10421 | 0.488 | 47.6 | Simple_repeat (159hits, 0.185%) | Ty1-INT Ty1/copia AleII (12 hits 0.115%) | AleII |  |
| CL61 | 2564250 | 10257 | 0.48 | 48.1 | LTR.Copia (130hits, 0.379%) | Ty1-RT Ty1/copia Maximus/SIRE (22 hits 0.214%) | Maximus/SIRE |  |
| CL62 | 2549500 | 10198 | 0.477 | 48.6 | LTR.Copia (**9730hits, 78.4%**) | Ty1-RH Ty1/copia Maximus/SIRE (4969 hits 48.7%) | Maximus/SIRE |  |
| CL63 | 2399500 | 9598 | 0.449 | 49 | LTR.Copia (**2259hits, 15.5%**) | Ty1-INT Ty1/copia Maximus/SIRE (1414 hits 14.7%) | Maximus/SIRE |  |
| CL64 | 2335250 | 9341 | 0.437 | 49.5 | LTR.Copia (243hits, 0.475%) | Ty1-RT Ty1/copia Maximus/SIRE (22 hits 0.236%) | Maximus/SIRE |  |
| CL65 | 2303750 | 9215 | 0.431 | 49.9 | LTR.Gypsy (151hits, 0.224%) | DTM-CD1 NA NA (8 hits 0.0868%) | NA |  |
| CL66 | 2288000 | 9152 | 0.428 | 50.3 | LTR.Copia (**3860hits, 35.7%**) | Ty1-RT Ty1/copia Maximus/SIRE (1970 hits 21.5%) | Maximus/SIRE |  |
| CL67 | 2229000 | 8916 | 0.417 | 50.7 | LTR.Copia (6hits, 0.0229%) | Ty1-RT Ty1/copia AleI/Retrofit (4 hits 0.0449%) | AleI/Retrofit |  |
| CL68 | 2158500 | 8634 | 0.404 | 51.2 | LTR.Caulimovirus (541hits, 1.56%) | Ty1-GAG Ty1/copia Ivana/Oryco (62 hits 0.718%) | Ivana/Oryco |  |
| CL69 | 2135250 | 8541 | 0.4 | 51.6 | Low_complexity (29hits, 0.0361%) | Ty3-INT Ty3/gypsy chromovirus (5 hits 0.0585%) | chromovirus |  |
| CL70 | 2073750 | 8295 | 0.388 | 51.9 | Low_complexity (279hits, 0.645%) | DTM-CD1 NA NA (205 hits 2.47%) | NA |  |
| CL71 | 2019750 | 8079 | 0.378 | 52.3 | LTR.Copia (567hits, 1.87%) | Ty1-GAG Ty1/copia Maximus/SIRE (679 hits 8.4%) | Maximus/SIRE |  |
| CL72 | 1967250 | 7869 | 0.368 | 52.7 | LTR.Gypsy (**1643hits, 14.6%**) | Ty3-INT Ty3/gypsy Ogre/Tat (906 hits 11.5%) | Ogre/Tat | 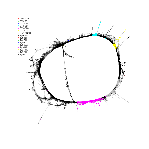 |
| CL73 | 1953000 | 7812 | 0.366 | 53.1 | Simple_repeat (239hits, 0.655%) | Ty3-INT Ty3/gypsy Ogre/Tat (24 hits 0.307%) | Ogre/Tat |  |
| CL74 | 1917250 | 7669 | 0.359 | 53.4 | LTR.Gypsy (**852hits, 8.91%**) | Ty3-INT Ty3/gypsy chromovirus (339 hits 4.42%) | chromovirus |  |
| CL75 | 1857750 | 7431 | 0.348 | 53.8 | Simple_repeat (58hits, 0.176%) | Ty3-INT Ty3/gypsy chromovirus (62 hits 0.834%) | chromovirus |  |
| CL76 | 1786000 | 7144 | 0.334 | 54.1 | LTR.Gypsy (**1457hits, 14.1%**) | Ty3-INT Ty3/gypsy Ogre/Tat (804 hits 11.3%) | Ogre/Tat |  |
| CL77 | 1754750 | 7019 | 0.329 | 54.4 | LTR.Gypsy (567hits, 1.52%) | LINE-RT NA NA (6 hits 0.0855%) | NA |  |
| CL78 | 1743000 | 6972 | 0.326 | 54.7 | DNA.MULE.MuDR (82hits, 0.516%) | DTM-CD1 NA NA (332 hits 4.76%) | NA |  |
| CL79 | 1735750 | 6943 | 0.325 | 55.1 | LTR.Gypsy (**940hits, 6.65%**) | Ty3-PROT Ty3/gypsy Athila (1347 hits 19.4%) | Athila |  |
| CL80 | 1701000 | 6804 | 0.319 | 55.4 | LTR.Gypsy (**4691hits, 57.8%**) | Ty3-INT Ty3/gypsy chromovirus (1525 hits 22.4%) | chromovirus |  |
| CL81 | 1691750 | 6767 | 0.317 | 55.7 | LTR.Copia (164hits, 0.492%) | DTM-CD1 NA NA (2 hits 0.0296%) | NA |  |
| CL82 | 1676250 | 6705 | 0.314 | 56 | LINE.RTE.BovB (**414hits, 4.65%**) | LINE-RT NA NA (251 hits 3.74%) | NA |  |
| CL83 | 1671250 | 6685 | 0.313 | 56.3 | LTR.Gypsy (**1352hits, 16%**) | Ty3-INT Ty3/gypsy chromovirus (509 hits 7.61%) | chromovirus |  |
| CL84 | 1649500 | 6598 | 0.309 | 56.6 | LTR.Gypsy (**880hits, 7.65%**) | Ty3-GAG Ty3/gypsy Athila (797 hits 12.1%) | Athila |  |
| CL85 | 1604000 | 6416 | 0.3 | 56.9 | LTR.Gypsy (**2439hits, 31.6%**) | Ty3-INT Ty3/gypsy chromovirus (970 hits 15.1%) | chromovirus |  |
| CL86 | 1487000 | 5948 | 0.278 | 57.2 | LTR.Copia (33hits, 0.156%) | Ty1-INT Ty1/copia AleI/Retrofit (1 hits 0.0168%) | AleI/Retrofit |  |
| CL87 | 1466750 | 5867 | 0.275 | 57.5 | LTR.Gypsy (104hits, 0.264%) | DTA-CD1 NA NA (1 hits 0.017%) | NA |  |
| CL88 | 1461250 | 5845 | 0.274 | 57.8 | LTR.Copia (**308hits, 3.33%**) |  |  |  |
| CL89 | 1394250 | 5577 | 0.261 | 58 | LTR.Gypsy (**1561hits, 22.4%**) | Ty3-INT Ty3/gypsy chromovirus (582 hits 10.4%) | chromovirus |  |
| CL90 | 1345500 | 5382 | 0.252 | 58.3 | LTR.Copia (**1042hits, 15.6%**) | Ty1-RT Ty1/copia Angela (313 hits 5.82%) | Angela |  |
| CL91 | 1294250 | 5177 | 0.242 | 58.5 | Low_complexity (24hits, 0.0482%) | DTA-CD1 NA NA (2 hits 0.0386%) | NA |  |
| CL92 | 1287000 | 5148 | 0.241 | 58.8 | LTR.Copia (485hits, 2.11%) | Ty1-RT Ty1/copia Maximus/SIRE (4 hits 0.0777%) | Maximus/SIRE |  |
| CL93 | 1262750 | 5051 | 0.236 | 59 | LTR.Copia (**3017hits, 44.6%**) | Ty1-RT Ty1/copia AleII (738 hits 14.6%) | AleII |  |
| CL94 | 1238500 | 4954 | 0.232 | 59.2 | LINE.L1 (60hits, 0.318%) | DHH-CD2 NA NA (3 hits 0.0606%) | NA |  |
| CL95 | 1160000 | 4640 | 0.217 | 59.5 | LTR.Gypsy (93hits, 0.415%) | Ty3-GAG Ty3/gypsy chromovirus (431 hits 9.29%) | chromovirus |  |
| CL96 | 1156500 | 4626 | 0.217 | 59.7 | DNA.PIF.Harbinger (72hits, 0.442%) | DTM-CD1 NA NA (3 hits 0.0649%) | NA |  |
| CL97 | 1134750 | 4539 | 0.212 | 59.9 | DNA.MULE.MuDR (82hits, 0.48%) | DTM-CD1 NA NA (768 hits 16.9%) | NA |  |
| CL99 | 1097500 | 4390 | 0.206 | 60.3 | rRNA (**2984hits, 63.2%**) |  |  |  |
| CL98 | 1100750 | 4403 | 0.206 | 60.1 | Low_complexity (152hits, 0.632%) | Ty1-RH Ty1/copia Maximus/SIRE (28 hits 0.636%) | Maximus/SIRE |  |
| CL101 | 1036000 | 4144 | 0.194 | 60.7 | Simple_repeat (169hits, 0.475%) | DTM-CD1 NA NA (4 hits 0.0965%) | NA |  |
| CL100 | 1037500 | 4150 | 0.194 | 60.5 | LTR.Gypsy (11hits, 0.0624%) | LINE-RT NA NA (2 hits 0.0482%) | NA |  |
| CL102 | 1028750 | 4115 | 0.193 | 60.9 | Simple_repeat (205hits, 1.43%) | Ty1-INT Ty1/copia AleII (8 hits 0.194%) | AleII |  |
| CL103 | 1027250 | 4109 | 0.192 | 61.1 | LTR.Gypsy (339hits, 1.46%) | DTC-CD1 NA NA (2 hits 0.0487%) | NA |  |
| CL105 | 994250 | 3977 | 0.186 | 61.4 | LTR.Gypsy (**391hits, 3.83%**) | Ty3-GAG Ty3/gypsy Athila (488 hits 12.3%) | Athila |  |
| CL104 | 995750 | 3983 | 0.186 | 61.3 | Low_complexity (69hits, 0.282%) | DTM-CD1 NA NA (2 hits 0.0502%) | NA |  |
| CL106 | 932000 | 3728 | 0.175 | 61.6 | LTR.Gypsy (242hits, 1.51%) | Ty3-RT Ty3/gypsy Athila (3 hits 0.0537%) | Athila |  |
| CL107 | 899500 | 3598 | 0.168 | 61.8 | LTR.Gypsy (**473hits, 6.36%**) | Ty3-INT Ty3/gypsy Athila (173 hits 4.81%) | Athila |  |
| CL108 | 885250 | 3541 | 0.166 | 62 | Low_complexity (148hits, 0.618%) | DTM-CD1 NA NA (313 hits 8.84%) | NA |  |
| CL109 | 864750 | 3459 | 0.162 | 62.1 | LTR.Copia (**2106hits, 51%**) | Ty1-RT Ty1/copia TAR (439 hits 12.7%) | TAR |  |
| CL110 | 856000 | 3424 | 0.16 | 62.3 | Low_complexity (38hits, 0.166%) | DTH-CD1 NA NA (2 hits 0.0584%) | NA | 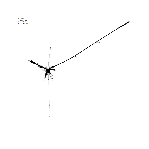 |
| CL111 | 846750 | 3387 | 0.159 | 62.4 | LTR.Copia (**930hits, 19.5%**) | Ty1-RT Ty1/copia Maximus/SIRE (268 hits 7.91%) | Maximus/SIRE |  |
| CL112 | 834250 | 3337 | 0.156 | 62.6 | LTR.Copia (**2422hits, 60.6%**) | Ty1-RT Ty1/copia Ivana/Oryco (592 hits 17.7%) | Ivana/Oryco |  |
| CL113 | 774250 | 3097 | 0.145 | 62.7 | Simple_repeat (**252hits, 3.94%**) | Ty3-GAG Ty3/gypsy chromovirus (2 hits 0.0646%) | chromovirus |  |
| CL114 | 765250 | 3061 | 0.143 | 62.9 | LTR.Copia (53hits, 0.837%) |  |  |  |
| CL115 | 756000 | 3024 | 0.142 | 63 | LTR.Gypsy (**1944hits, 56.2%**) | Ty3-INT Ty3/gypsy Athila (669 hits 22.1%) | Athila |  |
| CL116 | 732250 | 2929 | 0.137 | 63.2 | DNA.MULE.MuDR (206hits, 2.45%) | DTM-CD1 NA NA (727 hits 24.8%) | NA |  |
| CL117 | 722750 | 2891 | 0.135 | 63.3 | Low_complexity (103hits, 0.501%) | Ty3-INT Ty3/gypsy chromovirus (5 hits 0.173%) | chromovirus |  |
| CL118 | 710500 | 2842 | 0.133 | 63.4 | LINE.L1 (9hits, 0.102%) | LINE-RT NA NA (1 hits 0.0352%) | NA |  |
| CL121 | 686750 | 2747 | 0.129 | 63.8 | LTR.Gypsy (336hits, 1.58%) | LINE-ENDO NA NA (71 hits 2.58%) | NA |  |
| CL119 | 691500 | 2766 | 0.129 | 63.6 | LTR.Copia (**879hits, 24.1%**) | Ty1-RT Ty1/copia Maximus/SIRE (257 hits 9.29%) | Maximus/SIRE |  |
| CL120 | 691250 | 2765 | 0.129 | 63.7 | Low_complexity (56hits, 0.559%) | Ty3-RH Ty3/gypsy chromovirus (5 hits 0.181%) | chromovirus |  |
| CL122 | 663750 | 2655 | 0.124 | 63.9 | LINE.L1 (**571hits, 13.2%**) | LINE-RT NA NA (474 hits 17.9%) | NA | 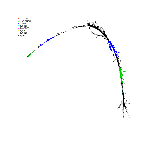 |
| CL123 | 657750 | 2631 | 0.123 | 64.1 | LTR.Gypsy (**294hits, 6.08%**) | Ty3-INT Ty3/gypsy chromovirus (305 hits 11.6%) | chromovirus |  |
| CL124 | 632250 | 2529 | 0.118 | 64.2 | Low_complexity (107hits, 0.552%) |  |  |  |
| CL125 | 622750 | 2491 | 0.117 | 64.3 | Simple_repeat (4hits, 0.0308%) | LINE-ENDO NA NA (1 hits 0.0401%) | NA |  |
| CL126 | 578500 | 2314 | 0.108 | 64.4 | rRNA (**2315hits, 99.1%**) |  |  |  |
| CL127 | 572500 | 2290 | 0.107 | 64.5 | DNA.CMC.EnSpm (**780hits, 26.7%**) | DTC-CD1 NA NA (555 hits 24.2%) | NA | 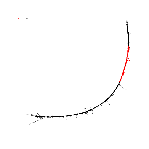 |
| CL128 | 553500 | 2214 | 0.104 | 64.6 | LTR.Gypsy (**182hits, 3.74%**) | Ty3-PROT Ty3/gypsy Athila (536 hits 24.2%) | Athila |  |
| CL129 | 533750 | 2135 | 0.1 | 64.7 | Simple_repeat (292hits, 1.77%) | Ty3-GAG Ty3/gypsy chromovirus (441 hits 20.7%) | chromovirus |  |
| CL130 | 510750 | 2043 | 0.096 | 64.8 | Low_complexity (304hits, 2.19%) |  |  |  |
| CL131 | 506250 | 2025 | 0.095 | 64.9 | Low_complexity (54hits, 0.32%) | DTM-CD1 NA NA (2 hits 0.0988%) | NA |  |
| CL132 | 505500 | 2022 | 0.095 | 65 | Low_complexity (112hits, 0.921%) | LINE-RT NA NA (2 hits 0.0989%) | NA |  |
| CL133 | 495500 | 1982 | 0.093 | 65.1 | DNA.PIF.Harbinger (**332hits, 13%**) | DTH-CD1 NA NA (450 hits 22.7%) | NA |  |
| CL134 | 477750 | 1911 | 0.089 | 65.2 | Low_complexity (84hits, 0.681%) |  |  |  |
| CL135 | 470750 | 1883 | 0.088 | 65.3 | LTR.Copia (**1272hits, 56.7%**) | Ty1-RT Ty1/copia Ivana/Oryco (359 hits 19.1%) | Ivana/Oryco |  |
| CL136 | 466750 | 1867 | 0.087 | 65.4 | Low_complexity (235hits, 1.6%) | DTA-CD1 NA NA (1 hits 0.0536%) | NA |  |
| CL137 | 456000 | 1824 | 0.085 | 65.4 | Low_complexity (59hits, 1.25%) | LINE-RT NA NA (4 hits 0.219%) | NA |  |
| CL138 | 448250 | 1793 | 0.084 | 65.5 | Low_complexity (**247hits, 5.03%**) | DTM-CD1 NA NA (11 hits 0.613%) | NA |  |
| CL139 | 442250 | 1769 | 0.083 | 65.6 | LTR.Copia (11hits, 0.131%) | Ty1-RT Ty1/copia Maximus/SIRE (5 hits 0.283%) | Maximus/SIRE |  |
| CL140 | 424500 | 1698 | 0.08 | 65.7 | Simple_repeat (41hits, 0.427%) | LINE-RT NA NA (101 hits 5.95%) | NA |  |
| CL141 | 414500 | 1658 | 0.078 | 65.8 | DNA.CMC.EnSpm (**587hits, 23.1%**) | DTC-CD1 NA NA (511 hits 30.8%) | NA |  |
| CL142 | 388250 | 1553 | 0.073 | 65.8 | Low_complexity (43hits, 0.296%) | Ty3-GAG Ty3/gypsy chromovirus (48 hits 3.09%) | chromovirus |  |
| CL143 | 371000 | 1484 | 0.07 | 65.9 | DNA.MULE.MuDR (**108hits, 3.79%**) | DTM-CD1 NA NA (98 hits 6.6%) | NA |  |
| CL144 | 339750 | 1359 | 0.064 | 66 | LTR.Copia (**737hits, 46.6%**) | Ty1-RT Ty1/copia TAR (152 hits 11.2%) | TAR |  |
| CL145 | 339250 | 1357 | 0.064 | 66 | LTR.Copia (**1297hits, 83.7%**) | Ty1-RT Ty1/copia AleI/Retrofit (430 hits 31.7%) | AleI/Retrofit |  |
| CL146 | 336000 | 1344 | 0.063 | 66.1 | LTR.Gypsy (21hits, 0.639%) | DTA-CD1 NA NA (1 hits 0.0744%) | NA |  |
| CL147 | 326000 | 1304 | 0.061 | 66.2 | DNA.CMC.EnSpm (**282hits, 17%**) | DTC-CD1 NA NA (178 hits 13.7%) | NA |  |
| CL149 | 312750 | 1251 | 0.059 | 66.3 | RC.Helitron (**384hits, 21%**) | DHH-CD1 NA NA (273 hits 21.8%) | NA |  |
| CL148 | 312750 | 1251 | 0.059 | 66.2 | LTR.Copia (**282hits, 16.4%**) | Ty1-RT Ty1/copia Angela (60 hits 4.8%) | Angela |  |
| CL150 | 303500 | 1214 | 0.057 | 66.3 | Low_complexity (52hits, 0.696%) | LINE-ENDO NA NA (1 hits 0.0824%) | NA |  |
| CL152 | 282000 | 1128 | 0.053 | 66.4 | LTR.Copia (**846hits, 63.1%**) | Ty1-RT Ty1/copia Ivana/Oryco (175 hits 15.5%) | Ivana/Oryco |  |
| CL151 | 284500 | 1138 | 0.053 | 66.4 | LTR.Copia (**257hits, 15.9%**) | Ty1-RT Ty1/copia Maximus/SIRE (102 hits 8.96%) | Maximus/SIRE |  |
| CL155 | 276500 | 1106 | 0.052 | 66.6 | LTR.Copia (2hits, 0.0506%) | Ty3-GAG Ty3/gypsy Ogre/Tat (105 hits 9.49%) | Ogre/Tat |  |
| CL153 | 280250 | 1121 | 0.052 | 66.5 | Low_complexity (**483hits, 5.31%**) |  |  |  |
| CL154 | 277750 | 1111 | 0.052 | 66.5 | Low_complexity (123hits, 1.97%) |  |  |  |
| CL157 | 247250 | 989 | 0.046 | 66.7 | Low_complexity (**390hits, 8.61%**) | LINE-RT NA NA (2 hits 0.202%) | NA |  |
| CL156 | 247500 | 990 | 0.046 | 66.6 | DNA.PIF.Harbinger (**240hits, 17.8%**) | DTH-CD1 NA NA (360 hits 36.4%) | NA | 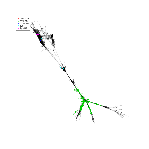 |
| CL158 | 238000 | 952 | 0.045 | 66.7 | LTR.Gypsy (5hits, 0.131%) | Ty3-INT Ty3/gypsy chromovirus (5 hits 0.525%) | chromovirus |  |
| CL159 | 236250 | 945 | 0.044 | 66.8 | DNA.MULE.MuDR (**55hits, 3%**) | DTM-CD1 NA NA (38 hits 4.02%) | NA | 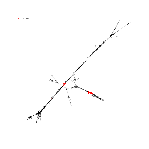 |
| CL160 | 227000 | 908 | 0.043 | 66.8 | Low_complexity (**106hits, 5.27%**) | Ty1-INT Ty1/copia AleII (1 hits 0.11%) | AleII |  |
| CL161 | 219250 | 877 | 0.041 | 66.9 | LTR.Gypsy (38hits, 0.878%) | Ty3-GAG Ty3/gypsy Athila (1 hits 0.114%) | Athila |  |
| CL162 | 218500 | 874 | 0.041 | 66.9 | Low_complexity (31hits, 0.476%) | DTH-CD1 NA NA (1 hits 0.114%) | NA |  |
| CL165 | 214000 | 856 | 0.04 | 67 | Simple_repeat (**176hits, 6.6%**) | Ty3-INT Ty3/gypsy Ogre/Tat (2 hits 0.234%) | Ogre/Tat |  |
| CL164 | 214500 | 858 | 0.04 | 67 | RC.Helitron (**292hits, 26.1%**) | DHH-CD2 NA NA (206 hits 24%) | NA |  |
| CL167 | 211750 | 847 | 0.04 | 67.1 | LTR.Gypsy (**84hits, 3.64%**) | Ty3-GAG Ty3/gypsy chromovirus (24 hits 2.83%) | chromovirus |  |
| CL166 | 212750 | 851 | 0.04 | 67.1 | LTR.Gypsy (**255hits, 22.8%**) | Ty3-INT Ty3/gypsy Ogre/Tat (137 hits 16.1%) | Ogre/Tat |  |
| CL163 | 215000 | 860 | 0.04 | 66.9 | Low_complexity (24hits, 0.5%) | DTH-CD1 NA NA (41 hits 4.77%) | NA |  |
| CL168 | 210000 | 840 | 0.039 | 67.1 | LTR.Gypsy (**586hits, 53.3%**) | Ty3-INT Ty3/gypsy chromovirus (213 hits 25.4%) | chromovirus |  |
| CL169 | 209500 | 838 | 0.039 | 67.2 | LTR.Copia (**388hits, 34.8%**) | Ty1-INT Ty1/copia AleII (130 hits 15.5%) | AleII |  |
| CL170 | 205750 | 823 | 0.038 | 67.2 | Low_complexity (**312hits, 5.05%**) |  |  |  |
| CL171 | 205250 | 821 | 0.038 | 67.3 | Low_complexity (**186hits, 3.85%**) |  |  |  |
| CL172 | 190000 | 760 | 0.036 | 67.3 | LTR.Gypsy (**264hits, 24.3%**) | Ty3-INT Ty3/gypsy Ogre/Tat (119 hits 15.7%) | Ogre/Tat |  |
| CL174 | 186750 | 747 | 0.035 | 67.4 | Simple_repeat (10hits, 0.147%) |  |  |  |
| CL173 | 189500 | 758 | 0.035 | 67.3 | LTR.Gypsy (**284hits, 25.2%**) | Ty3-INT Ty3/gypsy Ogre/Tat (130 hits 17.2%) | Ogre/Tat |  |
| CL175 | 186500 | 746 | 0.035 | 67.4 | LTR.Copia (3hits, 0.0874%) |  |  |  |
| CL176 | 183500 | 734 | 0.034 | 67.4 | DNA.PIF.Harbinger (**277hits, 26.3%**) | DTH-CD1 NA NA (517 hits 70.4%) | NA |  |
| CL177 | 175750 | 703 | 0.033 | 67.5 | LTR.Gypsy (**516hits, 63.1%**) | Ty3-INT Ty3/gypsy chromovirus (137 hits 19.5%) | chromovirus |  |
| CL178 | 170750 | 683 | 0.032 | 67.5 | Low_complexity (191hits, 2.99%) | Ty3-RT Ty3/gypsy chromovirus (2 hits 0.293%) | chromovirus |  |
| CL179 | 165250 | 661 | 0.031 | 67.5 | Low_complexity (19hits, 0.448%) |  |  |  |
| CL180 | 157250 | 629 | 0.029 | 67.6 | Low_complexity (5hits, 0.0967%) | DTH-CD1 NA NA (1 hits 0.159%) | NA |  |
| CL181 | 146000 | 584 | 0.027 | 67.6 | LTR.Copia (**532hits, 79.1%**) | Ty1-RT Ty1/copia Ivana/Oryco (108 hits 18.5%) | Ivana/Oryco |  |
| CL182 | 143250 | 573 | 0.027 | 67.6 | LTR.Copia (**386hits, 45.1%**) | Ty1-RH Ty1/copia Bianca (65 hits 11.3%) | Bianca |  |
| CL183 | 138000 | 552 | 0.026 | 67.6 | LTR.Copia (**512hits, 80.9%**) | Ty1-RT Ty1/copia Tork (131 hits 23.7%) | Tork |  |
| CL185 | 131000 | 524 | 0.025 | 67.7 | LTR.Copia (**108hits, 14.5%**) |  |  |  |
| CL184 | 132500 | 530 | 0.025 | 67.7 | Low_complexity (**63hits, 3.93%**) |  |  |  |
| CL190 | 126750 | 507 | 0.024 | 67.8 | RC.Helitron (**93hits, 13.5%**) | DHH-CD2 NA NA (124 hits 24.5%) | NA |  |
| CL187 | 129000 | 516 | 0.024 | 67.7 | LTR.Gypsy (**280hits, 39.1%**) | Ty3-INT Ty3/gypsy Ogre/Tat (198 hits 38.4%) | Ogre/Tat |  |
| CL188 | 127750 | 511 | 0.024 | 67.8 | LTR.Copia (**63hits, 6.63%**) | Ty1-PROT Ty1/copia Maximus/SIRE (37 hits 7.24%) | Maximus/SIRE |  |
| CL191 | 125750 | 503 | 0.024 | 67.8 | Low_complexity (6hits, 0.115%) |  |  |  |
| CL186 | 129500 | 518 | 0.024 | 67.7 | Low_complexity (23hits, 1.94%) | DTM-CD1 NA NA (3 hits 0.579%) | NA |  |
| CL189 | 127500 | 510 | 0.024 | 67.8 | Low_complexity (22hits, 0.479%) |  |  |  |
| CL192 | 125000 | 500 | 0.023 | 67.9 | RC.Helitron (**97hits, 11.9%**) | DTA-CD1 NA NA (1 hits 0.2%) | NA |  |
| CL193 | 124000 | 496 | 0.023 | 67.9 | LTR.Copia (13hits, 0.743%) |  |  |  |
| CL194 | 122000 | 488 | 0.023 | 67.9 | Low_complexity (14hits, 0.78%) | DHH-CD1 NA NA (1 hits 0.205%) | NA |  |
| CL195 | 120500 | 482 | 0.023 | 67.9 | Low_complexity (**113hits, 3.03%**) | DTC-CD1 NA NA (1 hits 0.207%) | NA |  |
| CL196 | 118750 | 475 | 0.022 | 67.9 | Low_complexity (14hits, 0.479%) |  |  |  |
| CL198 | 110250 | 441 | 0.021 | 68 | Low_complexity (9hits, 0.194%) | Ty1-GAG Ty1/copia Ivana/Oryco (1 hits 0.227%) | Ivana/Oryco |  |
| CL197 | 112250 | 449 | 0.021 | 68 | Low_complexity (16hits, 1.1%) | DTM-CD1 NA NA (1 hits 0.223%) | NA |  |
| CL200 | 105500 | 422 | 0.02 | 68 | LTR.Copia (17hits, 0.612%) | Ty1-GAG Ty1/copia Tork (1 hits 0.237%) | Tork |  |
| CL199 | 108000 | 432 | 0.02 | 68 | Low_complexity (43hits, 1.3%) |  |  |  |
| CL202 | 103750 | 415 | 0.019 | 68.1 | RC.Helitron (**152hits, 21.5%**) |  |  |  |
| CL206 | 99500 | 398 | 0.019 | 68.1 | LTR.Copia (**233hits, 33.4%**) | Ty1-RH Ty1/copia TAR (3 hits 0.754%) | TAR |  |
| CL204 | 101000 | 404 | 0.019 | 68.1 | Low_complexity (8hits, 0.256%) |  |  |  |
| CL203 | 103500 | 414 | 0.019 | 68.1 | Low_complexity (24hits, 1.5%) |  |  |  |
| CL201 | 104250 | 417 | 0.019 | 68 | Low_complexity (17hits, 1.4%) | DTM-CD1 NA NA (5 hits 1.2%) | NA |  |
| CL205 | 100750 | 403 | 0.019 | 68.1 | DNA.MULE.MuDR (**31hits, 3.64%**) | DTM-CD1 NA NA (80 hits 19.9%) | NA |  |
| CL212 | 93250 | 373 | 0.018 | 68.2 | LTR.Gypsy (3hits, 0.151%) |  |  |  |
| CL208 | 98500 | 394 | 0.018 | 68.2 | LTR.Gypsy (**220hits, 45.8%**) | Ty3-INT Ty3/gypsy chromovirus (80 hits 20.3%) | chromovirus |  |
| CL211 | 93500 | 374 | 0.018 | 68.2 | LTR.Copia (2hits, 0.153%) | LINE-ENDO NA NA (2 hits 0.535%) | NA |  |
| CL207 | 99000 | 396 | 0.018 | 68.2 | LTR.Copia (**207hits, 39%**) | Ty1-RT Ty1/copia AleII (62 hits 15.7%) | AleII |  |
| CL209 | 94500 | 378 | 0.018 | 68.2 | Low_complexity (6hits, 0.263%) |  |  |  |
| CL210 | 93500 | 374 | 0.018 | 68.2 | Low_complexity (23hits, 0.981%) | Ty3-RT Ty3/gypsy chromovirus (2 hits 0.535%) | chromovirus |  |
| CL213 | 92500 | 370 | 0.017 | 68.3 | Simple_repeat (7hits, 0.17%) | DTA-CD1 NA NA (2 hits 0.541%) | NA |  |
| CL217 | 88000 | 352 | 0.017 | 68.3 | LTR.Gypsy (1hits, 0.0477%) | Ty3-RT Ty3/gypsy chromovirus (1 hits 0.284%) | chromovirus |  |
| CL215 | 90750 | 363 | 0.017 | 68.3 | LTR.Copia (1hits, 0.109%) | Ty3-GAG Ty3/gypsy Ogre/Tat (38 hits 10.5%) | Ogre/Tat |  |
| CL216 | 88500 | 354 | 0.017 | 68.3 | Low_complexity (60hits, 2.54%) |  |  |  |
| CL214 | 92500 | 370 | 0.017 | 68.3 | Low_complexity (53hits, 2.81%) | DTM-CD1 NA NA (1 hits 0.27%) | NA |  |
| CL218 | 86250 | 345 | 0.016 | 68.3 | Simple_repeat (3hits, 0.186%) | LINE-RT NA NA (21 hits 6.09%) | NA |  |
| CL220 | 84750 | 339 | 0.016 | 68.4 | LTR.Gypsy (**167hits, 36.2%**) | Ty3-RT Ty3/gypsy Ogre/Tat (111 hits 32.7%) | Ogre/Tat |  |
| CL219 | 85750 | 343 | 0.016 | 68.4 | LINE.L1 (2hits, 0.273%) | Ty1-INT Ty1/copia AleII (1 hits 0.292%) | AleII |  |
| CL224 | 81750 | 327 | 0.015 | 68.4 | Simple_repeat (2hits, 0.286%) |  |  |  |
| CL230 | 77500 | 310 | 0.015 | 68.5 | LTR.Gypsy (**63hits, 13%**) | Ty3-PROT Ty3/gypsy Athila (86 hits 27.7%) | Athila |  |
| CL222 | 82250 | 329 | 0.015 | 68.4 | LTR.Gypsy (2hits, 0.146%) | Ty1-GAG Ty1/copia Ivana/Oryco (18 hits 5.47%) | Ivana/Oryco |  |
| CL223 | 82000 | 328 | 0.015 | 68.4 | LTR.Gypsy (**203hits, 51.2%**) | Ty3-INT Ty3/gypsy chromovirus (67 hits 20.4%) | chromovirus |  |
| CL229 | 78000 | 312 | 0.015 | 68.5 | LTR.Gypsy (**128hits, 32.7%**) | Ty3-INT Ty3/gypsy chromovirus (104 hits 33.3%) | chromovirus |  |
| CL221 | 83000 | 332 | 0.015 | 68.4 | LTR.Copia (**298hits, 69.2%**) | Ty1-RH Ty1/copia Tork (64 hits 19.3%) | Tork |  |
| CL226 | 81250 | 325 | 0.015 | 68.5 | LTR.Copia (**213hits, 51.5%**) | Ty1-GAG Ty1/copia Ivana/Oryco (46 hits 14.2%) | Ivana/Oryco |  |
| CL228 | 78250 | 313 | 0.015 | 68.5 | LTR.Copia (1hits, 0.069%) |  |  |  |
| CL225 | 81500 | 326 | 0.015 | 68.5 | Low_complexity (32hits, 1.52%) |  |  |  |
| CL231 | 77250 | 309 | 0.015 | 68.5 | DNA.hAT.Ac (5hits, 0.313%) | Ty1-INT Ty1/copia AleII (1 hits 0.324%) | AleII |  |
| CL227 | 79500 | 318 | 0.015 | 68.5 | DNA (**32hits, 3.22%**) | Ty3-GAG Ty3/gypsy chromovirus (1 hits 0.314%) | chromovirus |  |
| CL232 | 76750 | 307 | 0.014 | 68.6 | LTR.Copia (**250hits, 65.2%**) | Ty1-RH Ty1/copia Ivana/Oryco (66 hits 21.5%) | Ivana/Oryco |  |
| CL235 | 73000 | 292 | 0.014 | 68.6 | LTR.Copia (12hits, 0.492%) | LINE-RT NA NA (1 hits 0.342%) | NA |  |
| CL234 | 73250 | 293 | 0.014 | 68.6 | Low_complexity (9hits, 0.365%) |  |  |  |
| CL233 | 73500 | 294 | 0.014 | 68.6 | DNA.PIF.Harbinger (**114hits, 29.9%**) | DTH-CD1 NA NA (175 hits 59.5%) | NA |  |
| CL236 | 72750 | 291 | 0.014 | 68.6 | DNA.MULE.MuDR (**25hits, 3.33%**) | DTM-CD1 NA NA (19 hits 6.53%) | NA |  |
| CL237 | 72500 | 290 | 0.014 | 68.6 | DNA.MULE.MuDR (**17hits, 3.37%**) | DTM-CD1 NA NA (11 hits 3.79%) | NA |  |
| CL238 | 70000 | 280 | 0.013 | 68.6 | Low_complexity (**42hits, 7.06%**) |  |  |  |
| CL239 | 67250 | 269 | 0.013 | 68.7 | LINE.L1 (16hits, 0.748%) | Ty1-INT Ty1/copia Maximus/SIRE (1 hits 0.372%) | Maximus/SIRE |  |
| CL240 | 67250 | 269 | 0.013 | 68.7 | DNA.hAT.Ac (**58hits, 9.09%**) |  |  |  |
| CL244 | 65250 | 261 | 0.012 | 68.7 | Simple_repeat (**15hits, 3.55%**) |  |  |  |
| CL248 | 63250 | 253 | 0.012 | 68.8 | LTR.Gypsy (**132hits, 43.4%**) | Ty3-RT Ty3/gypsy chromovirus (57 hits 22.5%) | chromovirus |  |
| CL242 | 65750 | 263 | 0.012 | 68.7 | LTR.Gypsy (**116hits, 36.1%**) | Ty3-RT Ty3/gypsy chromovirus (49 hits 18.6%) | chromovirus |  |
| CL245 | 65250 | 261 | 0.012 | 68.7 | LTR.Copia (**190hits, 56.2%**) | Ty1-RT Ty1/copia AleII (55 hits 21.1%) | AleII |  |
| CL243 | 65750 | 263 | 0.012 | 68.7 | Low_complexity (39hits, 2.7%) | DTH-CD1 NA NA (1 hits 0.38%) | NA |  |
| CL247 | 64000 | 256 | 0.012 | 68.8 | Low_complexity (27hits, 2.83%) | Ty3-GAG Ty3/gypsy chromovirus (1 hits 0.391%) | chromovirus |  |
| CL249 | 62750 | 251 | 0.012 | 68.8 | Low_complexity (15hits, 0.759%) |  |  |  |
| CL246 | 65000 | 260 | 0.012 | 68.7 | DNA.MULE.MuDR (**31hits, 5.43%**) | DTM-CD1 NA NA (109 hits 41.9%) | NA |  |
| CL241 | 66250 | 265 | 0.012 | 68.7 | DNA.hAT.Ac (**47hits, 7.41%**) | DTA-CD1 NA NA (149 hits 56.2%) | NA |  |
| CL252 | 60500 | 242 | 0.011 | 68.8 | RC.Helitron (**93hits, 21.2%**) | DHH-CD1 NA NA (124 hits 51.2%) | NA |  |
| CL260 | 58500 | 234 | 0.011 | 68.9 | RC.Helitron (**104hits, 35.8%**) | DHH-CD2 NA NA (123 hits 52.6%) | NA |  |
| CL257 | 59250 | 237 | 0.011 | 68.9 | LTR.Gypsy (**72hits, 16.8%**) | Ty3-INT Ty3/gypsy Ogre/Tat (97 hits 40.9%) | Ogre/Tat |  |
| CL251 | 61000 | 244 | 0.011 | 68.8 | LTR.Copia (**76hits, 18.7%**) | Ty1-GAG Ty1/copia Bianca (9 hits 3.69%) | Bianca |  |
| CL254 | 60000 | 240 | 0.011 | 68.8 | LTR.Copia (**53hits, 13.4%**) | Ty1-GAG Ty1/copia Ivana/Oryco (73 hits 30.4%) | Ivana/Oryco |  |
| CL250 | 61000 | 244 | 0.011 | 68.8 | LTR.Copia (**134hits, 40.1%**) | Ty1-INT Ty1/copia AleII (55 hits 22.5%) | AleII |  |
| CL256 | 59500 | 238 | 0.011 | 68.9 | Low_complexity (64hits, 2.37%) | LINE-ENDO NA NA (6 hits 2.52%) | NA |  |
| CL253 | 60500 | 242 | 0.011 | 68.8 | Low_complexity (28hits, 1.63%) | Ty3-INT Ty3/gypsy Athila (1 hits 0.413%) | Athila |  |
| CL258 | 59250 | 237 | 0.011 | 68.9 | Low_complexity (21hits, 2.2%) | DTM-CD1 NA NA (14 hits 5.91%) | NA |  |
| CL261 | 57500 | 230 | 0.011 | 68.9 | Low_complexity (16hits, 1.16%) | DTM-CD1 NA NA (116 hits 50.4%) | NA |  |
| CL262 | 56500 | 226 | 0.011 | 68.9 | LINE.L1 (**31hits, 10.1%**) | LINE-RT NA NA (44 hits 19.5%) | NA |  |
| CL255 | 59750 | 239 | 0.011 | 68.8 | DNA.hAT.Ac (**53hits, 9.76%**) | DTA-CD1 NA NA (119 hits 49.8%) | NA |  |
| CL259 | 59000 | 236 | 0.011 | 68.9 | DNA.hAT.Ac (**176hits, 39.1%**) | DTH-CD1 NA NA (2 hits 0.847%) | NA |  |
| CL263 | 55750 | 223 | 0.01 | 68.9 | Simple_repeat (**50hits, 10.1%**) |  |  |  |
| CL265 | 54250 | 217 | 0.01 | 69 | LTR.Copia (**145hits, 48.6%**) | Ty1-RT Ty1/copia AleII (42 hits 19.4%) | AleII |  |
| CL264 | 55000 | 220 | 0.01 | 68.9 | NA |  |  |  |
| CL266 | 54000 | 216 | 0.01 | 69 | NA |  |  |  |
