# Supplementary material for: The nature and genomic landscape of repetitive DNA classes in Chrysanthemum nankingense shows recent genomic changes
Source: Ann Bot. 2022 May 27;131(1):215–28. doi: 10.1093/aob/mcac066 (PMC9904347; doi:10.1093/aob/mcac066)
Supplement: mcac066_suppl_Supplementary_Table_S2 [file mcac066_suppl_supplementary_table_s2.docx]

Zhang et al. The nature and genomic landscape of repetitive DNA classes in *Chrysanthemum nankingense* shows recent genomic changes

Supplementary Table S2: k-mer assembly statistics in *Chrysanthemum nankingense*.

Table S2-1 Assembly of 16-merGTE50000 identified in *Chrysanthemum nankingense*.

| Statistics | Unused Reads | All Contigs | Contigs >= 100 bp | Contigs >= 1000 bp |
| --- | --- | --- | --- | --- |
| Number of | 9 | 39 | 0 | 0 |
| Min Length (bp) | 16 | 17 | 0 | 0 |
| Median Length (bp) |  | 21 | 0 | 0 |
| Mean Length (bp) | 16 | 23 | 0 | 0 |
| Max Length (bp) | 16 | 39 | 0 | 0 |
| N50 Length (bp) |  | 22 | 0 | 0 |
| Number of contigs >=N50 |  | 16 | 0 | 0 |
| Length Sum (bp) | 144 | 904 | 0 | 0 |

GTE: greater than or equal.

Table S2-2 Assembly of 16-merGTE10000 identified in *Chrysanthemum nankingense*.

| Statistics | Unused Reads | All Contigs | Contigs >= 100 bp | Contigs >= 1000 bp |
| --- | --- | --- | --- | --- |
| Number of | 474 | 3255 | 173 | 1 |
| Min Length (bp) | 16 | 17 | 100 | 1972 |
| Median Length (bp) |  | 26 | 137 | 1972 |
| Mean Length (bp) | 16 | 39 | 172 | 1972 |
| Max Length (bp) | 16 | 1972 | 1972 | 1972 |
| N50 Length (bp) |  | 45 | 163 | 1972 |
| Number of contigs >=N50 |  | 693 | 55 | 1 |
| Length Sum (bp) | 7584 | 127956 | 29798 | 1972 |

GTE: greater than or equal.

Table S2-3 Assembly of 64-merGTE10000 identified in *Chrysanthemum nankingense*.

| Statistics | Unused Reads | All Contigs | Contigs >= 100 bp | Contigs >= 1000 bp |
| --- | --- | --- | --- | --- |
| Number of | 1 | 20 | 8 | 0 |
| Min Length (bp) | 64 | 65 | 102 | 0 |
| Median Length (bp) |  | 91 | 123 | 0 |
| Mean Length (bp) | 64 | 99 | 133 | 0 |
| Max Length (bp) | 64 | 211 | 211 | 0 |
| N50 Length (bp) |  | 102 | 129 | 0 |
| Number of contigs >=N50 |  | 8 | 4 | 0 |
| Length Sum (bp) | 64 | 1987 | 1066 | 0 |

GTE: greater than or equal.

Table S2-4 Assembly of 64-merGTE1000 identified in *Chrysanthemum nankingense*.

| Statistics | Unused Reads | All Contigs | Contigs >= 100 bp | Contigs >= 1000 bp |
| --- | --- | --- | --- | --- |
| Number of | 21 | 2525 | 1710 | 16 |
| Min Length (bp) | 64 | 65 | 100 | 1027 |
| Median Length (bp) |  | 130 | 189 | 1361 |
| Mean Length (bp) | 64 | 209 | 270 | 2995 |
| Max Length (bp) | 64 | 24844 | 24844 | 24844 |
| N50 Length (bp) |  | 283 | 337 | 24844 |
| Number of contigs >=N50 |  | 486 | 380 | 1 |
| Length Sum (bp) | 1344 | 528547 | 463199 | 47923 |

GTE: greater than or equal.

Table S2-5 Assembly of 128-merGTE1000 identified in *Chrysanthemum nankingense*.

| Statistics | Unused reads | All contigs | Contigs >= 128 bp | Contigs >= 1000 bp |
| --- | --- | --- | --- | --- |
| Number | 9 | 229 | 229 | 11 |
| Min Length (bp) | 128 | 129 | 129 | 1066 |
| Median Length (bp) |  | 240 | 240 | 1903 |
| Mean Length (bp) | 128 | 406 | 406 | 2517 |
| Max Length (bp) | 128 | 6202 | 6202 | 6202 |
| N50 Length (bp) |  | 538 | 538 | 3327 |
| Number of contigs >=N50 |  | 40 | 40 | 3 |
| Length Sum (bp) | 1152 | 93017 | 93017 | 27696 |

GTE: greater than or equal.
