# Supplementary material for: The nature and genomic landscape of repetitive DNA classes in Chrysanthemum nankingense shows recent genomic changes
Source: Ann Bot. 2022 May 27;131(1):215–28. doi: 10.1093/aob/mcac066 (PMC9904347; doi:10.1093/aob/mcac066)
Supplement: mcac066_suppl_Supplementary_Table_S3 [file mcac066_suppl_supplementary_table_s3.docx]

Zhang et al. The nature and genomic landscape of repetitive DNA classes in *Chrysanthemum nankingense* shows recent genomic changes

| **Name** | **Contig size (bp)** | **Estimated fragment size (bp)** | **Forward primer** | **Reverse primer** |
| --- | --- | --- | --- | --- |
| CL1Contig456 | 2280 | 412 | CTCGCTGGGACATAACGACC | CACCCCCAGTTAGGTAGTGC |
| CL2Contig586 | 2027 | 555 | AGACACCTGAGCAACGAAGC | TGAACACACAATACACACACACG |
| CL4Contig81 | 1246 | 501 | TAACACAACGTCGCCTACCC | GTGAAAGGTGCTCATCATCCG |
| CL6Contig83 | 1523 | 501 | CGAGGCACATATTGAGGGGG | CTAGCTCAGTCTCAGTCCGC |
| CL7Contig38 | 1476 | 533 | TGCTACGCGTAAGGTGATCC | AAAACGCACCCAGAATCACG |
| CL9Contig290 | 1599 | 493 | CCACTCAGTTCCAAGTCCCG | AGTGAGTTTCATAGCCCCTACC |
| CL10Contig254 | 2208 | 597 | TGGCTGACGTCGTATCATGG | TCCCGTACATTGCATCTCGG |
| CL35Contig138 | 5487 | 511 | GGCACAATTCTATAACCCTTGCG | ACGGAGTGTACACCCATAAGG |
| CL72Contig70 | 2375 | 425 | AGGCTAGGGGTTTTGCTTCC | GGTTAAGTCCCCGCAGTACC |
| CL110Contig5 | 1919 | 353 | ACACGTTTCCTCACTTTAAACACC | GTGACTATTGATCATCCAATGGCC |
| CL122Contig35 | 1960 | 203 | GTGTAACCATCGGGACCAGG | GTTGCAGGTGCTTTTGTTGC |
| CL127Contig9 | 8168 | 472 | AGGCATCACAGTGTATGGCC | TTTGTTCCCCGGTCATTGGG |
| CL156Contig21 | 1815 | 419 | AATAGTAAATGCACGCGCCG | GTCCGAAAGAATGGCAAGGC |
| CL159Contig16 | 2735 | 214 | TTGGAACCAGGAAGCTCAGC | GGTACATGATGGTTTTTGTGCC |
| Telomere | 134 | 116 | TAAACCCTAAACCCTAAACC | TTTAGGGTTTAGGGTTTAGG |
| 5S | 363 | 198 | CCCATCAGAACTCCGCAGTT | GCTGTTCCGGTCGTTTTGAC |
| 45S | 5842 | 1985 | ACCACATCCAAGGAAGGCAG | CGAGCAACAGCACGAAACAA |
| *Copia* |  | 270 | ACNGCNTTYYTNCAYGG | ARCATRTCRTCNACRTA |
| *Gypsy* |  | 420 | MRNATGTGYGTNGAYTAYMG | YKNWSNGGNTAYCAYCARAT |

Supplementary Table S3: Primer sequences used in this study.
