# Supplementary material for: The nature and genomic landscape of repetitive DNA classes in Chrysanthemum nankingense shows recent genomic changes
Source: Ann Bot. 2022 May 27;131(1):215–28. doi: 10.1093/aob/mcac066 (PMC9904347; doi:10.1093/aob/mcac066)
Supplement: mcac066_suppl_Supplementary_Table_S4 [file mcac066_suppl_supplementary_table_s4.docx]

Zhang et al. The nature and genomic landscape of repetitive DNA classes in *Chrysanthemum nankingense* shows recent genomic changes

Supplementary Table S4: Proportion of repetitive DNA sequences in the *Chrysanthemum nankingense* genome.

| Item | Subfamily | Data from Song *et al.*, 2018 *) | Repeat Explorer **) | LTR-Retriever ***)  All elements (intact elements) |
| --- | --- | --- | --- | --- |
| SINE |  | 0.02% |  |  |
| LINE |  | 1.31% | 0.84% |  |
| LTR-retroelements | Total | 47.10% | 49.53% | 55.71% (4.26%) |
|  | *Gypsy* | 21.54% | 21.72% †) | 11.98% (0.54%) |
|  | *Copia* | 25.40% | 26.48% †) | 18.88% (1.69%) |
|  | *Caulimovirus* |  | 1.33% |  |
|  | unknown |  |  | 24.85% (2.03%) |
| DNA transposon |  | 3.18% | 1.57% |  |
|  | RC.Helitron |  | 1.02 |  |
| Satellite |  | 0.32% |  |  |
|  | rRNA |  | 1.13% |  |
| Simple repeat |  | 1.02% | 5.78% |  |
| Low complexity |  | 0.11% | 9.07% |  |
| Other |  | 0.94% | 0.02% |  |
| Unknown |  | 17.62% |  |  |
| Total |  | 69.58% | 68.96% |  |

*) From their Supplementary Table S9; who searched for known repetitive sequences in the *C. nankingense* genome sequences using a cross-match program with a Repbase-derived RepeatMasker library with RepeatModeler.

**) RepeatExplorer clusters were identified as listed in Supplementary Table S1 and genome proportions added together.

***) Scaffolds were downloaded from the Song *et al.* 2018 assembly and then LTR-retroelements identified using LTR-retriever (see M&M)

†) 16.18% *LTR.Copia* and 23.03% *LTR.Gypsy* were classified to lineages, see Supplementary data Figure S1.
